# Carboplatin versus two doses of cisplatin in combination with gemcitabine in the treatment of advanced non-small-cell lung cancer: Results from a British Thoracic Oncology Group randomised phase III trial

**DOI:** 10.1016/j.ejca.2017.05.037

**Published:** 2017-09

**Authors:** David Ferry, Lucinda Billingham, Hugh Jarrett, David Dunlop, Penella J. Woll, Marianne Nicolson, Riyaz Shah, Joyce Thompson, James Spicer, D. Muthukumar, Geraldine Skailes, Pauline Leonard, A.D. Chetiyawardana, Paula Wells, Conrad Lewanski, Barbara Crosse, Michelle Hill, Piers Gaunt, Kenneth O'Byrne

**Affiliations:** a Royal Wolverhampton NHS Trust, Wolverhampton, UK; b Cancer Research UK Clinical Trials Unit, University of Birmingham, Birmingham, UK; c Beatson West of Scotland Cancer Centre, Glasgow, UK; d Weston Park Hospital, University of Sheffield, Sheffield, UK; e Aberdeen Royal Infirmary, Aberdeen, UK; f Kent Oncology Centre, Maidstone Hospital, Maidstone, UK; g Heart of England NHS Foundation Trust, Birmingham, UK; h King's College London, Guy's Hospital, London, UK; i Colchester General Hospital, Colchester, UK; j Lancashire Teaching Hospitals NHS Foundation Trust, Preston, UK; k Whittington Health NHS Trust, Whittington Hospital, London, UK; l Queen Elizabeth Hospital, Birmingham, UK; m Barts Health NHS Trust, London, UK; n Imperial College Healthcare, Charing Cross Hospital, London, UK; o Calderdale and Huddersfield NHS Foundation Trust, Huddersfield, UK; p St James' Hospital, Dublin, Ireland

**Keywords:** Non-small-cell lung cancer, Carboplatin, Cisplatin, Gemcitabine, Randomised phase III trial, Quality of life

## Abstract

**Background:**

Platinum-based combination chemotherapy is standard treatment for the majority of patients with advanced non-small-cell lung cancer (NSCLC). The trial investigates the importance of the choice of platinum agent and dose of cisplatin in relation to patient outcomes.

**Methods:**

The three-arm randomised phase III trial assigned patients with chemo-naïve stage IIIB/IV NSCLC in a 1:1:1 ratio to receive gemcitabine 1250 mg/m^2^ on days 1 and 8 of a 3-week cycle with cisplatin 80 mg/m^2^ (GC80) or cisplatin 50 mg/m^2^ (GC50) or carboplatin AUC6 (GCb6) for a maximum of four cycles. Primary outcome measure was survival time, aiming to test for a difference between treatment arms and also assess non-inferiority with pre-defined margin selected as hazard ratio (HR) of 1.2. Secondary outcome measures included response rate, adverse events and quality of life (QoL).

**Findings:**

The trial recruited 1363 patients. Survival time differed significantly across the three treatment arms (p = 0.046) with GC50 worst with median 8.2 months compared to 9.5 for GC80 and 10.0 for GCb6. HRs (adjusted) for GC50 compared to GC80 was 1.13 (95% confidence interval [CI] 0.99–1.29) and for GC50 compared to GCb6 was 1.23 (95% CI: 1.08–1.41). GCb6 was significantly non-inferior to GC80 (HR = 0.93, upper limit of one-sided 95% CI 1.04). Adjusting for QoL did not change the findings. Best objective response rates were 29% (GC80), 20% (GC50) and 27% (GCb6), p < 0.007. There were more dose reductions and treatment delays in the GCb6 arm and more adverse events (60% with at least one grade 3–4 compared to 43% GC80 and 30% GC50).

**Interpretation:**

In combination with gemcitabine, carboplatin at AUC6 is not inferior to cisplatin at 80 mg/m^2^ in terms of survival. Carboplatin was associated with more adverse events and not with better quality of life. Cisplatin at the lower dose of 50 mg/m^2^ has worse survival which is not compensated by better quality of life.

**ClinicalTrials.gov identifier:**

NCT00112710.

**EudraCT Number:**

2004-003868-30.

**Cancer Research UK trial identifier:**

CRUK/04/009.

## Highlights

• Large randomised phase III trial in advanced non-small cell lung cancer is reported.
• Platinum-based chemotherapy remains standard first-line treatment for majority.
• Trial provides definitive evidence-base for choice of platinum in this setting.
• Cisplatin 50 mg/m^2^ gives inferior survival, not compensated by better quality of life.
• Carboplatin AUC6 is not inferior to cisplatin 80 mg/m^2^ in terms of survival time and is not associated with better quality of life.

## 1 Introduction

Lung cancer is the leading cause of cancer death worldwide [1] and is responsible for more than 20% of cancer deaths in the United Kingdom [2]. Non-small-cell lung cancer (NSCLC) accounts for more than 80% of lung cancers and poor outcomes are driven by the fact that the vast majority present at clinic with advanced disease [3]. This paper reports a large randomised phase III trial in advanced NSCLC, set up by the British Thoracic Oncology Group (the BTOG2 trial), to provide definitive evidence to inform choice of standard first-line treatments. Early presentations of the results from the trial have already influenced clinical practice and this paper provides the final conclusive published evidence.

There is continued uncertainty about the optimal first-line chemotherapy for patients with advanced NSCLC and hence clinical practice remains variable. Platinum-based combination chemotherapy was firmly established following a meta-analysis of eight cisplatin randomised trials [4] which was later confirmed by an updated meta-analysis of 16 further trials [5] but there was ongoing ambiguity about whether cisplatin or carboplatin gave better patient outcomes. This was driven by conflicting trial results, in particular emerging results from an influential UK trial giving evidence that carboplatin with gemcitabine gave better survival than cisplatin (low dose 50 mg/m^2^) combined with mitomycin and ifosfamide [6] and a meta-analysis of five trials suggesting that in combination with third generation drugs, such as gemcitabine and taxanes, cisplatin gave better survival and higher radiological response rates than carboplatin [7].

In addition, there was uncertainty about the preferred dose of cisplatin due to a lack of definitive evidence, with practitioners in the UK more inclined to opt for the lower dose of 50 mg/m^2^ every three weeks [6] than counterparts in Europe and the United States which considered 75–100 mg/m^2^ as standard [8], [9]. The cisplatin burden of intravenous hydration and inpatient administration together with the toxicity of emesis, neuropathy and perception of poor tolerance led many clinicians to adopt carboplatin as the preferred option. Carboplatin however is largely renally cleared and must be correctly dosed according to glomerular filtration rate (GFR) [10] and measurement of GFR with 51-Cr-EDTA is cumbersome and expensive. Even when dosed optimally, carboplatin causes more severe neutropenia and thrombocytopaenia than cisplatin [11]. The BTOG2 trial aimed to resolve this cisplatin versus carboplatin debate.

A large randomised trial in advanced NSCLC in the USA had shown no differences in response rate or survival for platinum combinations with gemcitabine, paclitaxel or docetaxel [8]. At the time of trial set-up, the most commonly used companion drug for platinum in the UK was gemcitabine, so this was adopted for the trial for all types of histology. It was not until 2008 that evidence arose to show that pemetrexed was a marginally superior companion drug to gemcitabine in non-squamous NSCLC [12]. However, with the focus of the BTOG2 trial on platinum choice, the trial remains relevant for all types of histology. The other key changes in standard of care is that patients with epidermal growth factor receptor (EGFR) mutations or anaplastic lymphoma kinase (ALK) gene rearrangements will receive tyrosine kinase inhibitors as standard first-line treatment as per European and USA guidelines [13], [14]. However this only affects a very small proportion of all NSCLC patients in the UK, 8% and 2%, respectively [15]. Recent results from a phase III trial [16] have lead to the additional option of an immune-checkpoint inhibitor in the first-line setting for a limited number (likely to be around 15% in real world practice) of patients with high PD-L1 expression. Thus platinum-based chemotherapy remains the standard of care and our trial results remain relevant for the majority of patients.

This paper reports the final results from the BTOG2 trial, which evaluates two doses of cisplatin compared with carboplatin in combination with gemcitabine as first-line treatment for advanced NSCLC to determine which gives the most benefit to patients. The trial has the major added strength of assessing long-term quality of life alongside survival in all participants.

## 2 Methods

### 2.1 Study design

BTOG2 was a three-arm randomised phase III clinical trial recruiting patients from 78 hospitals in the United Kingdom and Ireland. Ethics approval for the trial protocol (ultimately Version 4) was obtained from West Midlands Research Ethics Committee and local institutional review boards and ethical committees in accordance with national and international guidelines.

### 2.2 Patients

Eligible patients had histologically or cytologically confirmed NSCLC with radiologically verified stage IIIB/IV disease not amenable to potentially curative treatment, with no clinically apparent brain metastases. Patients had no known concomitant or previous malignancy likely to interfere with protocol treatment or trial evaluations and no prior chemotherapy or radiotherapy. They were at least 18 years old with a World Health Organization performance status (PS) score of 0–2 and life expectancy of >12 weeks, adequate organ and haematologic function and no severe acute or chronic medical condition that would have impaired the ability to participate in the study or the interpretation of results. Pregnant and breast-feeding women were excluded and those with reproductive potential were required to use effective methods of contraception. All patients gave written informed consent.

### 2.3 Randomisation and masking

Eligible patients were randomly assigned 1:1:1 to receive gemcitabine 1250 mg/m^2^ days 1 and 8 of a 3-week cycle plus on day 1 cisplatin 80 mg/m^2^ (GC80) or cisplatin 50 mg/m^2^ (GC50) or carboplatin AUC6 (GCb6) for a maximum of four cycles. Treatment allocation was by telephone to the central randomisation service at the Cancer Research UK Clinical Trials Unit at University of Birmingham. Randomisation was stratified by stage of disease (IIIB versus IV) and PS (0 versus 1 versus 2) and balanced within treatment centres. Treatment was allocated to patients sequentially using an in-house validated minimisation algorithm.

### 2.4 Procedures

Protocol drugs were delivered intravenously either as inpatient or outpatient, according to local practice. The estimate of GFR used in the Calvert formula, both for carboplatin dosing and to determine eligibility (creatinine clearance >60 mL/min), used the Wright equation (the version with creatinine kinase correction and either enzymatic or Jaffe serum creatinine measurement) which is equivalent to 51-Cr-EDTA clearance [17]. To ensure correct dosing we provided an Excel spreadsheet calculator (Supplementary Material Appendix 1). Also to ensure optimal and pragmatic hydration for cisplatin patients, all participating centres complied with the BTOG2 recommended schedule (Supplementary Material Appendix 2). Dose adjustments and cycle delays (up to 3 weeks) were permitted in the event of toxicity with protocol-specific recommendations. Patients were to be treated for four cycles or until disease progression or unacceptable toxicity as per physician judgement. Standard anti-emetics were 5 d of a 5-HT3 antagonist plus dexamethasone 4 mg twice daily or, after day 8 gemcitabine, oral domperidone 20 mg up to four times daily as required.

Pre-treatment evaluation included: medical history (including cancer history and prior anti-cancer treatments), clinical examination (including PS, blood pressure, ECG), laboratory analyses (complete blood count and coagulation tests, blood chemistry, creatinine clearance with Wright equation) and tumour assessment by appropriate imaging techniques with measurable lesions being a requirement for the trial. Computed tomography scan was performed at baseline, after two cycles (6 weeks), four cycles (12 weeks) and where possible was repeated at time of withdrawing from treatment. Response was assessed with Response Evaluation Criteria in Solid Tumours (RECIST) 1.0 [18] locally but there was no requirement for confirmation of response. Patients had chest x-rays during treatment and follow-up in accordance with local practice. Adverse events according to National Cancer Institute-Common Terminology Criteria for Adverse Events (NCI-CTCAE) version 3.0 [19] were recorded at every clinic visit. Follow-up data were collected at standard post-treatment clinic visits at approximately monthly intervals. Quality of life questionnaires were administered by research nurses prior to randomisation and on day 1 of each treatment cycle prior to receiving treatment and at every follow-up visit (typically monthly). They were completed independently by patients.

### 2.5 Outcomes

The primary outcome was survival time measured in whole days from randomisation to death from any cause, with censoring at date of last follow-up for those with no death date at time of database lock (28th May 2014). Quality of life was an important secondary outcome measure. Eligibility criteria required the patient to be willing and able to complete quality of life questionnaires which included three validated instruments: the generic and lung cancer instruments developed by the European Organisation for Research and Treatment of Cancer EORTC QLQ-C30 [20] and QLQ-LC13 [21] and the standardised instrument to measure utilities developed by the EuroQol Group, EQ-5D [22]. Other secondary outcome measures included: best overall response (based on RECIST 1.0) [18]; dose intensity of chemotherapy (calculated for each drug as the mean of dose intensities for each cycle received, given by actual versus expected dose per day); proportion of cycles given as an outpatient; incidence of adverse events (graded ≥2 according to NCI-CTCAE version 3.0) [19] and costs and cost-effectiveness (to be reported in a separate paper).

### 2.6 Statistical analysis

The survivor function for each treatment arm is estimated using Kaplan–Meier method from which medians and 1-year rates are reported with Greenwood's formula used for 95% confidence intervals (CIs). All treatment arms could be considered ‘standard practice’ so the primary analysis tests the null hypothesis of no difference between the three treatment arms initially using a log-rank test as specified in the protocol but supplemented by Cox regression model that accounts for stratification factors of stage and PS as the more recently preferred analytical approach [23]. Regression coefficients from the model provide estimates of hazard ratios (HR) with two-sided 95% CIs to compare treatment arms. As planned, the analysis also tests for non-inferiority between treatment arms, permissible under the closed test procedure [24], using one-sided 95% CI for HRs with non-inferiority inferred when the entire CI falls within the non-inferiority region pre-defined by a margin for the HR of 1.2. All analyses of the primary outcome measure were based on an intention-to-treat (ITT) principle.

At the design stage, sample size calculations were based on the primary outcome measure of survival time. For a log-rank test comparing three treatment arms, 400 deaths were required per arm to enable a difference in median survival of 2 months (7 versus 9) to be detected between any of the three arms with 90% power. This is equivalent to an absolute difference in 1-year survival rates of the order of 35% versus 45% and an HR of 0.78. Assuming an accrual period of 3 years and follow-up period of 1 year, it was estimated that 450 patients per arm would be need to achieve the required number of events giving total target recruitment of 1350. With 400 deaths per arm and using a one-sided 95% CI there is 80% power to detect non-inferiority based on a pre-defined non-inferiority margin for the HR of 1.2 or absolute difference in median survival of 6 weeks.

Detailed analysis of the longitudinal quality of life (QoL) data will be reported in a separate paper but quality-adjusted survival time is reported here using a method called the integrated quality-survival product [25]. Survival time, represented by the Kaplan–Meier function, is adjusted for QoL using a step function of the utility measure from EQ-5D, representing the mean of all responses from participants still alive at each point across time. The analysis is based on ITT and restricted to 12 months from trial entry.

Objective response rates are compared using an ITT analysis and chi-square test. Analysis of the remaining secondary outcome measures was based on the per-protocol population defined as those who received at least one cycle of their assigned treatment. Dose intensity for platinum and gemcitabine are compared using one-way analysis of variance. Proportion of chemotherapy delivery days as an outpatient rather than inpatient are compared using a chi-square test. Incidence of each type of adverse event is reported descriptively with a chi-square test pre-selected in the statistical analysis plan to compare incidence of at least one grade 3 or 4 adverse event during treatment.

An independent Data Monitoring Committee reviewed interim data annually to ensure patient safety. There were no formal stopping rules. The trial was registered on the EU Clinical Trials Register with EudraCT number 2004-003868-30.

### 2.7 Role of the funding source

The trial was sponsored by University of Birmingham and run by the Cancer Research UK Clinical Trials Unit located there. Funding came from Cancer Research UK supplemented by an educational grant from Eli Lilly and Company Ltd. The trial was initiated and conducted independently by the trial investigators. The funder had no role in trial design, data collection, data analysis, data interpretation or writing of the report. The corresponding author had full access to all the data in the trial and had final responsibility for the decision to submit for publication.

## 3 Results

**Fig. 1:**
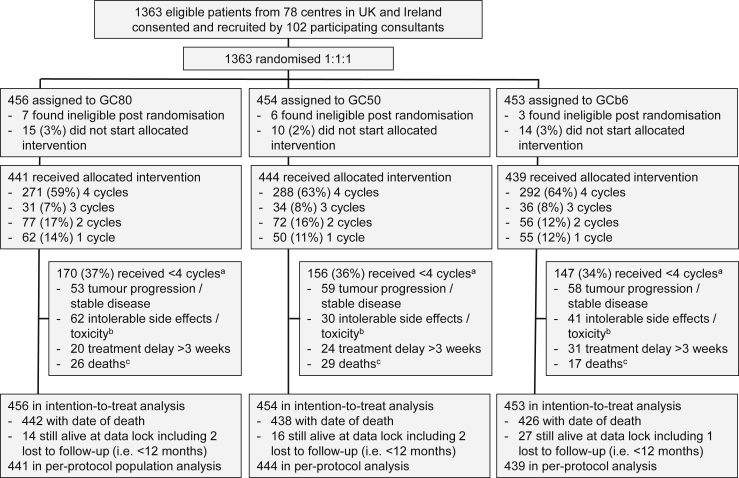
: Trial profile. ^a^Multiple reasons were recorded and frequencies reporting the top four reasons are given here; ^b^includes grade 3 or 4 non-haematological or symptomatic grade 4 haematological; ^c^died within 28 d of day 1 of the last cycle received.

Between April 2005 and November 2009, 1363 patients were randomised, 456 to GC80, 454 to GC50 and 453 to GCb6 (Fig. 1). Patient characteristics and disease history at baseline were well balanced across the treatment arms (Table 1). The median age for patients in the trial was 63 years (range 29–83) with predominance of males (62%) and PS 1 (60%) but also including 8% PS 2. Stage IV disease was most common (68%) with 38% having adenocarcinoma histology, 35% squamous cell, 3% large cell and the remaining 23% unspecified. Post-randomisation, 16 patients were found to be ineligible with the most common reason being biochemical measures found to be marginally outside of the required range, but half received protocol treatment and all are included in the ITT analysis.

**Table 1:** Baseline patient and disease characteristics.

Characteristic	GC80 (N = 456)	GC50 (N = 454)	GCb6 (N = 453)
Male	286 (63%)	291 (64%)	268 (59%)
Female	170 (37%)	163 (36%)	185 (41%)
**Age**			
Median	63	63	63
IQ range	57.5–68	57–69	57–68
Range	30–79	32–82	29–83
**Stage**^a^			
IIIB	146 (32%)	145 (32%)	144 (32%)
IV	310 (68%)	309 (68%)	309 (68%)
**WHO PS**^a^			
0	146 (32%)	146 (32%)	145 (32%)
1	275 (60%)	274 (60%)	274 (60%)
2	35 (8%)	34 (8%)	34 (8%)
**Prior surgery**			
No	427 (97%)	416 (95%)	412 (95%)
Yes	15 (3%)	20 (5%)	23 (5%)
Not reported	14	18	18
**Histology**			
Squamous	149 (33%)	152 (33%)	156 (34%)
Adenocarcinoma	169 (37%)	156 (34%)	182 (40%)
Large cell	14 (3%)	12 (3%)	13 (3%)
Unspecified	124 (27%)	134 (30%)	102 (23%)
**BSA**			
N	440	440	441
Median	1.82	1.84	1.82
IQ range	1.68–1.98	1.67–1.97	1.67–1.98
Range	1.35–2.26	1.26–2.45	1.28–2.49
**Target lesion size (mm)**			
N	425	421	430
Median	82	82	83
IQ range	57–117	52–125	56–121
Range	4–553	5–389	10–420

WHO PS, World Health Organization performance status; IQ, interquartile; BSA, body surface area.

a Indicates stratification factors.

Treatment delivery details within each of the treatment arms is summarised in Fig. 1. The majority of patients (62%) received the planned four cycles of treatment and this was balanced across treatment arms. 39 patients (2.9%) did not start treatment due to clinical deterioration. The most common reason for early withdrawal from treatment in all arms is tumour progression or stable disease. Grade 3 or 4 non-haematological toxicity and general intolerable side-effects from treatment was most common in GC80. Symptomatic grade 4 haematological toxicity was most common in GCb6. Across cycles, dose reductions and cycle delays occurred most in GCb6 and least in GC50 (Supplementary Material Appendix 3) which translated into a significant difference in dose intensity of platinum and gemcitabine across treatment arms (Table 2; p < 0.0001 for both). Dose intensity was lowest for GCb6 but medians on all treatment arms were at least 80%. Dosing of carboplatin used the Wright equation which gave prescribed doses on average 10% more (interquartile range 4%–17% and range −14% to 51%) than the Cockcroft–Gault formula (Supplementary Material Appendix 4).

**Table 2:** Comparison of secondary outcome measures across treatment arms.

Secondary outcome measure	GC80	GC50	GCb6	p-value
**Best overall response rate**				
Number (%) of patients with reported CR or PR	132/456 (29%)	92/454 (20%)	123/453 (27%)	0.007
**Dose intensity for platinum**				
N	423	430	406	
Median	94%	98%	83%	<0.0001
Interquartile range	81%–99%	90%–100%	72%–97%	
Range	22%–116%	19%–113%	19%–118%	
Number (%) of patients ≥90%	256 (61%)	327 (76%)	146 (36%)	
**Dose intensity for gemcitabine**				
N	419	429	423	
Median	87%	94%	80%	
Interquartile range	74–98%	83–99%	68–94%	<0.0001
Range	40–116%	43–107%	32–106%	
Number (%) of patients ≥90%	192 (46%)	260 (61%)	127 (30%)	
**Proportion of chemotherapy delivery days as an outpatient**				
N	441	444	439	
Median	87.5%	100%	100%	<0.0001
Number (%) of patients 100%	207 (47%)	246 (55%)	281 (64%)	
**Adverse events**				
Number (%) of patients with at least 1 grade 3 or 4 adverse event reported during treatment	190/441 (43%)	133/444 (30%)	263/439 (60%)	<0.0001

CR, complete response; PR, partial response.

The incidence of key adverse events at grades 2–4 are shown in Table 3. Of the total 6802 events reported across the 4284 patient-cycles received, the majority (77%) were grade 2, 15% grade 3 and 4% grade 4 (3% unspecified grade). Hearing loss and tinnitus were predominantly reported for GC80 but at a low rate with patient-cycle incidence rate of 7% and only 2% reported as grade 3 or 4. As expected, rates of nausea and vomiting were higher in GC80 compared to GC50 but absolute differences were small in levels of grade 3–4 (2.8% versus 0.6% for nausea and 2.9% versus 0.2% for vomiting). GCb6 was associated with the highest rates of myelosuppression but with low rates for grade 3–4; anaemia 4%, neutropenia 16% and thrombocytopaenia 10%. Fatigue was no different between GC80 and GCb6 with grade 3–4 patient-cycle incidence rates of 3.7% versus 3.4%, respectively. Documented infections occurred at a similar low rate on the three arms. Overall, the patient incidence rates for at least one reported grade 3 or 4 adverse event differed significantly across the three treatment arms (Table 2; p < 0.0001) with greatest of 60% on GCb6 compared with 30% on GC50.

**Table 3:** Comparison of treatment arms in terms of key adverse events (i.e. patient-cycle grade 2–4 incidence ≥10% and/or difference ≥5%).

Adverse event	Grade	Patient-cycles with adverse events of the specified grade			Treated patients with at least one adverse event of specified grade		
		GC80 (N = 1393)	GC50 (N = 1448)	GCb6 (N = 1443)	GC80 (N = 441)	GC50 (N = 444)	GCb6 (N = 439)
Nausea	≥2	262 (19%)	118 (8%)	151 (11%)	161 (37%)	85 (19%)	108 (25%)
	≥3	39 (2.8%)	9 (0.6%)	19 (1.3%)	34 (7.7%)	8 (1.8%)	16 (3.6%)
Vomiting	≥2	178 (13%)	59 (4%)	77 (5%)	121 (27%)	45 (10%)	58 (13%)
	≥3	34 (2.9%)	3 (0.2%)	12 (0.8%)	29 (6.6%)	2 (0.5%)	10 (2.3%)
Constipation	≥2	166 (12%)	134 (9%)	144 (10%)	121 (27%)	90 (20%)	101 (23%)
	≥3	5 (0.3%)	5 (0.3%)	5 (0.3%)	5 (1.1%)	5 (1.1%)	5 (1.1%)
Dyspnoea	≥2	136 (10%)	105 (7%)	180 (13%)	90 (20%)	87 (20%)	119 (27%)
	≥3	24 (1.7%)	27 (1.9%)	29 (2.0%)	23 (5.2%)	25 (5.6%)	23 (5.2%)
Anaemia	≥2	173 (12%)	154 (11%)	419 (29%)	110 (25%)	103 (23%)	233 (53%)
	≥3	13 (0.9%)	10 (0.7%)	58 (4.0%)	10 (2.3%)	9 (2.0%)	49 (11.2%)
Neutropenia	≥2	155 (11%)	105 (7%)	384 (27%)	109 (25%)	77 (17%)	238 (54%)
	≥3	74 (5.3%)	49 (3.4%)	227 (15.7%)	60 (13.6%)	40 (9.0%)	163 (37.1%)
Thrombocytopaenia	≥2	67 (5%)	32 (2%)	213 (15%)	55 (12%)	28 (6%)	138 (31%)
	≥3	32 (2.3%)	13 (0.9%)	144 (10.0%)	29 (6.6%)	12 (2.7%)	103 (23.5%)
Fatigue	≥2	379 (27%)	326 (23%)	389 (27%)	222 (50%)	193 (43%)	225 (51%)
	≥3	51 (3.7%)	29 (2.0%)	49 (3.4%)	43 (9.8%)	25 (5.6%)	42 (9.6%)
Ototoxicity	≥2	92 (7%)	44 (3%)	17 (1%)	66 (15%)	28 (6%)	10 (2%)
	≥3	18 (1.3%)	2 (0.1%)	0 (0%)	15 (3.4%)	1 (0.2%)	0 (0%)

The proportion of patients treated in the outpatient setting is significantly greater for GCb6 (Table 2; p < 0.0001) with 64% of patients receiving all their cycles as an outpatient compared to only 47% of patients on GC80. Best response rate to treatment significantly differed between the three treatment arms (p = 0.007; Table 2) with comparable rates for GC80 (29%) and GCb6 (27%) and a lower rate for GC50 (20%).

**Fig. 2:**
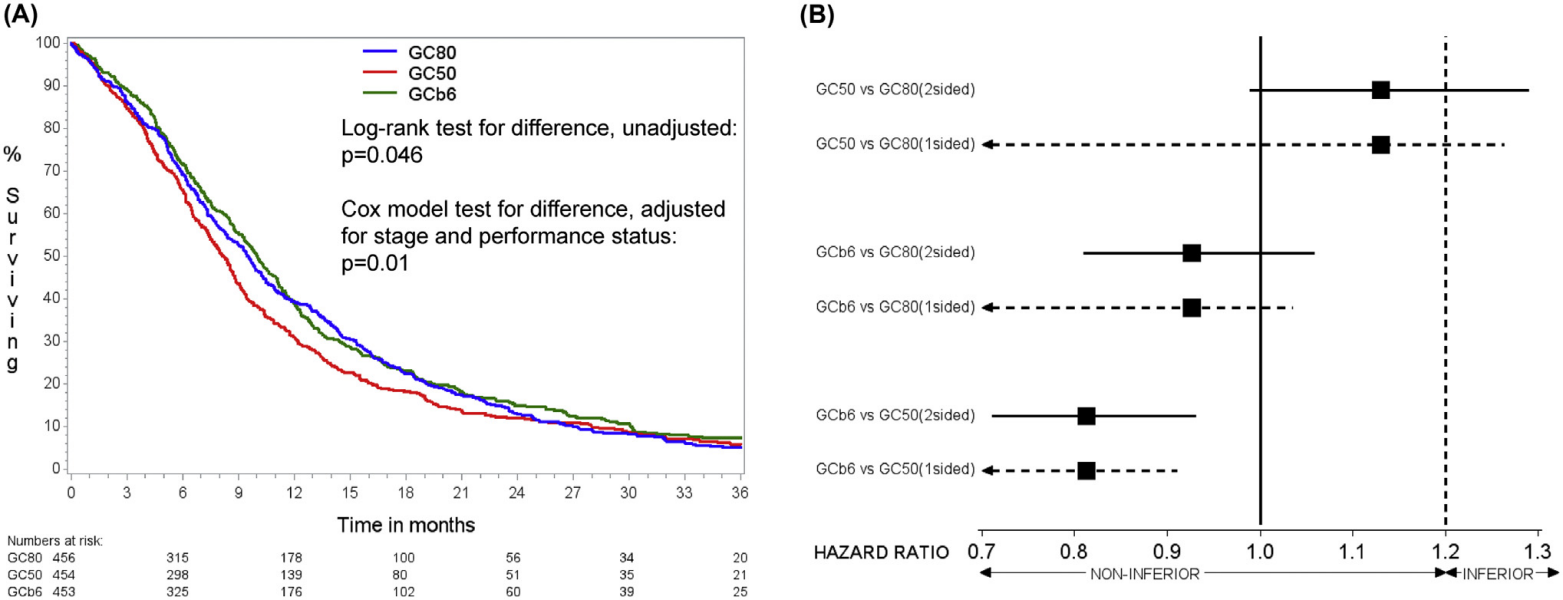
(A) Kaplan–Meier survivor functions for each treatment group. (B) Pairwise comparisons of survival showing HRs (adjusted for stage and performance status) with two-sided 95% CIs (solid line) for assessment of difference (compare either end against HR = 1) and one-sided 95% CIs (dashed line) for assessment of non-inferiority (compare upper values against HR = 1.2).

At the time of data lock (28th May 2014) there were 1306 deaths. Of the 57 patients still alive, median follow-up time was 29 months with maximum of 80 and including 5 patients lost to follow-up within 12 months of entry. Eleven patients died within 28 d of randomisation without starting treatment and 80 patients died during treatment i.e. within 28 d of day 1 of their last cycle of chemotherapy (29, 31 and 20 on GC80, GC50 and GCb6, respectively). Kaplan–Meier estimates of survival (Fig. 2A, Table 4) show that GC50 had the worst survival time with median 8.2 months whilst GC80 and GCb6 were comparable with medians of 9.5 and 10.0 months, respectively. This difference between treatment arms was statistically significant (unadjusted and adjusted p-values 0.046 and 0.01, respectively). Paired comparisons of treatments (Fig. 2B) show that this difference is driven primarily by the inferior survival of GC50 compared to the other two arms (adjusted HR for GC50 versus GC80 of 1.13, two-sided 95% CI: 0.99–1.29 and adjusted HR for GC50 versus GCb6 of 1.23, two-sided 95% CI: 1.08–1.41). Furthermore, GCb6 was found to be significantly non-inferior to GC80 with adjusted HR = 0.93 (unadjusted 0.94) and upper limit of one-sided 95% CI as 1.04 (unadjusted 1.05).

**Table 4:** Comparison of survival time and quality-adjusted survival time across treatment arms.

Summary statistic	GC80	GC50	GCb6
One year survival rates (95% CIs)	39% (35%–44%)	31% (27%–35%)	39% (34%–43%)
Median survival time in months (95% CIs)	9.5 (8.4–10.3)	8.2 (7.4–8.7)	10.0 (9.2–10.8)
Mean quality-adjusted survival time in months (within 12 months) (95% CIs)	6.0 (5.7–6.3)	5.6 (5.2–5.9)	6.1 (5.8–6.5)

**Fig. 3:**
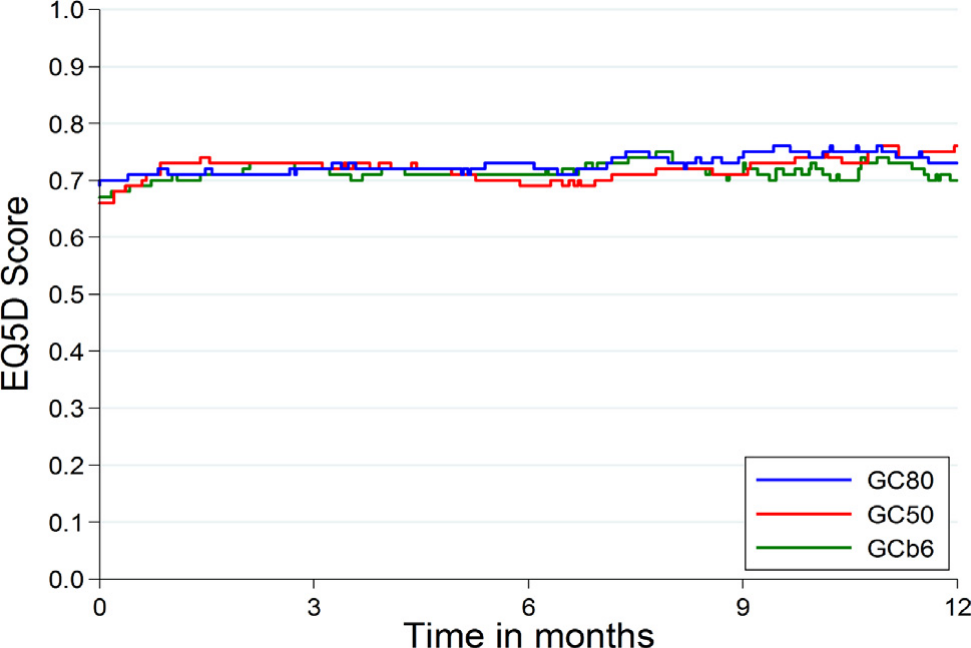
Mean EQ-5D utility score over 12 months (represented as a step function joining the means of all patients still alive at each observed death time in the trial).

Overall quality of life over time, as measured by the EQ-5D utility measure (where 0 represents quality equivalent to death and 1 represents ‘perfect health’) is relatively constant over time and similar in all three treatment groups (Fig. 3). Quality-adjusted survival time shows the same pattern of results across treatment arms as overall survival time (Table 4) with GC50 worst and GC80 and GCb6 comparable.

## 4 Discussion

This randomised phase III trial compared the effects of two doses of cisplatin and carboplatin AUC6 in combination with gemcitabine in a treatment naive population of patients with advanced NSCLC. The trial was undertaken in an era before widespread testing for activating mutations of EGFR and ALK, histological differentiation and maintenance chemotherapy were standard practice and the newly introduced immune-checkpoint inhibitor for tumours with high PD-L1 expression. With these changes in clinical practice only relevant for small selected subgroups of patients, platinum-based combination chemotherapy remains the standard first-line treatment for the majority of patients with this disease.

The trial included what could be regarded as the highest possible safe dose of carboplatin, using the Wright equation [17] considered to be equivalent to the Calvert formula [10]. For cisplatin, the trial selected the highest dose in common use (80 mg/m^2^) compared with the lower dose commonly prescribed in the UK (50 mg/m^2^). The principal conclusion is that GCb6 is not inferior to GC80 in terms of survival time. This key result runs counter to all previous meta-analysis [7], [26], [27] which concluded cisplatin was superior to carboplatin. Such analyses have strengths and correctly identified that cisplatin-based treatment was beneficial in NSCLC, but they also have limitations, especially when details such as dose, dose intensity and how doses of carboplatin were calculated are significant variables. These factors could have contributed to decreased effectiveness of carboplatin in the meta-analysis. This drug has predominant renal excretion and is no longer prescribed on a body surface area formula but on the Calvert formula [10]. Central to using this formula is estimating GFR. In the original work, 51-Cr-EDTA methodology was used and widely regarded as the gold standard. However this can be closely approximated by the Wright formula [17]. This is clearly superior to the Cockcroft–Gault formula, which is easy to compute but underestimates GFR by an average of 10%. Many previous clinical trials allowed sites to vary the method of GFR estimation or used low doses such as AUC5 and Cockcroft–Gault GFR estimation [26]. Having delivered the maximum safe dose of carboplatin combined with gemcitabine, we found that this drug is not inferior in survival terms to the highest reasonable dose of cisplatin (80 mg/m^2^).

Having conducted the largest ever randomised trial comparing carboplatin with cisplatin in NSCLC, we have high resolution adverse event and quality of life data. When first introduced, cisplatin had a deserved reputation for often severe emesis, renal damage and neuropathy [28]. Improvements in anti-emetics, 5-HT3 and NK1 receptor antagonists [29] and better hydration have attenuated these effects such that although GC80 produced more grade 3–4 vomiting (2.9%) than carboplatin (0.8%), the difference is not clinically significant. Also the trial has enabled a reduction in the hydration schedule duration for cisplatin, such that it is easy to deliver cisplatin as a day case. However, carboplatin does produce worse myelosuppresion, neutropenia and thrombocytopaenia but with no significant impact on infections or deaths on treatment. The data on survival, response rates and toxicity are comparable to other large randomised trials. In the trial [12] of gemcitabine plus cisplatin at 75 mg/m^2^ (GC75) versus pemetrexed plus cisplatin (PC75) the median survival for GC75 was 10.3 (BTOG2 9.5), response rate was 31% (BTOG2 29%) and febrile neutropenia 3.7% (infection rate 3% in BTOG2).

In many solid tumours, sequences of chemotherapy regimens have produced dramatic improvement. The best example is possibly colorectal cancer where overall survival has increased over the last 20 years from around 10–12 months to 25–30 months [30]. Instead of only 5FU/folinic acid these patients have combinations of 5FU, oxalipaltin and irinotecan often combined with the antiangiogenic bevacizumab. To RAS wild type patients, anti-EGFR antibodies are also given. NSCLC has been slower to develop sequential therapy, but for adenocarcinoma patients maintenance therapy with pemetrexed immediately after first line cisplatin-gemcitabine and taxanes after carboplatin-gemcitabine were each shown to be beneficial. The recent KEYNOTE-024 trial [16], which included comparable patients to BTOG2 including both squamous and non-squamous histologies and selected for high expression of PD-L1, demonstrated that the immune-checkpoint inhibitor pembrolizumab improves progression-free survival time and overall survival time in the first-line setting in comparison to chemotherapy and may become the first-line treatment of choice for selected patients. Of the 1934 patients screened, 1653 had samples that could be evaluated for PD-L1, 500 (30%) had high expression and 305 were randomised into the trial demonstrating that this treatment was an option for only 15% of the screened population. Because patients are not cured by first-line checkpoint inhibitors, for those who are eligible for this option, it is important that optimal platinum-based combination chemotherapy follows to produce best results for patients. This illustrates that chemotherapy practice and principles will remain the same, but sequencing for some may change. Optimising all aspects of anti-cancer treatments is essential, especially the doses and schedules of chemotherapy drugs which may impact survival and the BTOG2 trial contributes important data in this regard.

In summary, the BTOG2 trial provides definitive evidence on the choice of platinum to partner with a second drug in standard first-line chemotherapy for advanced NSCLC and provides for the first time comprehensive quality of life data to support decision-making. Carboplatin dosed at AUC6 using the Wright equation (or Calvert equation) gives non-inferior survival to cisplatin dosed at 80 mg/m^2^ and cisplatin at the lower dose of 50 mg/m^2^ has worse survival which is not compensated by better quality of life.

## Funding

Funding was from Cancer Research UK supplemented by an educational grant from Eli Lilly and Company Ltd. The trial was initiated and conducted independently by the trial investigators.

## Conflict of interest statement

David Ferry reports personal fees from Eli Lilly and Company during the conduct of the study. Lucinda Billingham reports an educational grant paid to the University of Birmingham from Eli Lilly and Company during the conduct of the study and personal fees from Eli Lilly and Company, Astra Zeneca, Pfizer, Roche and Celgene outside the submitted work. Hugh Jarrett reports an educational grant paid to University of Birmingham from Eli Lilly and Company during the conduct of the study. Riyaz Shah reports free drug for the trial supplied by Eli and Lilly and Company during the conduct of the study and personal fees from Eli Lilly and Company outside the submitted work. Pauline Leonard reports personal fees from Eli Lilly and Company, Amgen, Bristol-Myers Squibb, Pfizer, Teva and Otsuka outside of the submitted work. Conrad Lewanski reports personal fees from Eli Lilly and Company, Roche and Astra Zeneca outside the submitted work. Kenneth O'Byrne has received advisory board and/or speaker bureau and/or meeting travel/registration support from Bristol-Myers Squibb, MSD, Eli Lilly and Company, Boehringer-Ingelheim, Pfizer, Novartis, Roche-Genentech and Astra Zeneca. Other authors have nothing to disclose.
