# Supplementary material for: Carboplatin versus two doses of cisplatin in combination with gemcitabine in the treatment of advanced non-small-cell lung cancer: Results from a British Thoracic Oncology Group randomised phase III trial
Source: Eur J Cancer. 2017 Sep;83:302–12. doi: 10.1016/j.ejca.2017.05.037 (PMC5597318; doi:10.1016/j.ejca.2017.05.037)

**Supplementary Material**

**Appendix 1: Excel spreadsheet calculator for carboplatin dosing**

| **BTOG2 Trial** |  |
| --- | --- |
| **Calculation of BSA, GFR and Carboplatin Dose** |  |
| **Using Wright Equation with Jaffe Serum Creatinine** |  |
| **See Appendix 6 of Protocol** |  |
|  |  |
| **Please enter data into the yellow shaded squares (tab between)** |  |
| **Subcalculations will automatically appear in the grey squares** |  |
| **The required values will automatically appear in the red squares** |  |
|  |  |
|  |  |
| CK (Creatine Kinase in units per litre): |  |
| A = 570 x Ln(CK) = |  |
|  |  |
| Age (in years): |  |
| B = 40 x Age = |  |
|  |  |
| Y = 4520 + A - B = |  |
|  |  |
| Sex (if female enter 1 or if male enter 0): |  |
| Z = 1 - (0.15 x Sex) = |  |
|  |  |
| Weight (in Kg) |  |
| Height (in cm) |  |
| **BSA (Dubois Body Surface Area in square metres) =** |  |
| calculated as 0.007184*(Weight^0.425)*(Height^0.725) |  |
|  |  |
| Numerator = Y x Z x BSA = |  |
|  |  |
| JSC (Jaffe Serum Creatinine in umol per litre): |  |
|  |  |
| **GFR (in ml/min) = Numerator / JSC =** |  |
| W = GFR + 25 = |  |
|  |  |
| **Carboplatin Dose (mg) = 6 x W =** |  |
|  |  |

**Appendix 2: Recommended hydration regimen for cisplatin delivery as specified in the BTOG2 protocol (changes to this schema were only permitted by prior approval of the Chief Investigator)**

|  | **Time Elapsed(hours)** | **Drug** | **Fluid** |
| --- | --- | --- | --- |
| **DAY 1** | 0 | Bolus 5-HT_3_ antagonist | **-** |
|  |  | Dexamethasone 8mg iv | - |
|  | 0 – 2 |  | N saline 1 litre |
|  | 2 – 2.5 | Gemcitabine 1250mg/m^2^ | N saline 0.25 litre |
|  | 2.5 – 3 | Mannitol 20% solution iv | 0.2 litre |
|  | 3 – 4 | Cisplatin 50 or 80mg/m^2^ | N saline 0.5 litre |
|  | 4 – 5 | 20 mmol KCl | N saline 1 litre |
|  | 5 – 6 | 1g Mg SO_4_ | 5% dextrose 1 litre |
| DAY 8 | 0 | Dexamethasone 4mg iv | - |
|  | 0 – 0.5 | Gemcitabine 1250mg/m^2^ | N saline 0.25 litre |

**Appendix 3: Dose reductions and cycle delays in protocol treatment delivery for those who received at least one cycle**

|  | **GC80** | **GC50** | **GCb6** |
| --- | --- | --- | --- |
| **Number with ≥1 dose reduction**  % of patients receiving each cycle who experienced dose reductions:  Cycle 1^a^  Cycle 2  Cycle 3  Cycle 4 | **57% (250/441)**  15% (68/441)  38% (143/379)  23% (69/302)  24% (65/271) | **38% (167/444)**  12% (53/444)  21% (83/394)  19% (60/322)  17% (50/288) | **74% (327/439)**  15% (67/439)  62% (236/384)  40% (130/328)  43% (125/292) |
| **Number with ≥1 cycle delay**  % of patients receiving each cycle who experienced cycle delay:  Cycle 1^a^  Cycle 2  Cycle 3  Cycle 4 | **51% (223/441)**  7% (31/441)  40% (151/379)  27% (81/302)  26% (71/271) | **48% (214/444)**  5% (24/444)  36% (141/394)  28% (91/322)  22% (62/288) | **61% (266/439)**  5% (22/439)  54% (208/384)  29% (95/328)  33% (96/292) |

^a^ reductions and delays relate only to day 8 gemcitabine

**Appendix 4: Comparison of actual carboplatin doses using Wright formula versus Cockcroft-Gault formula (N=409; 44 unavailable for analysis due to missing data; green line represents line of equality)**


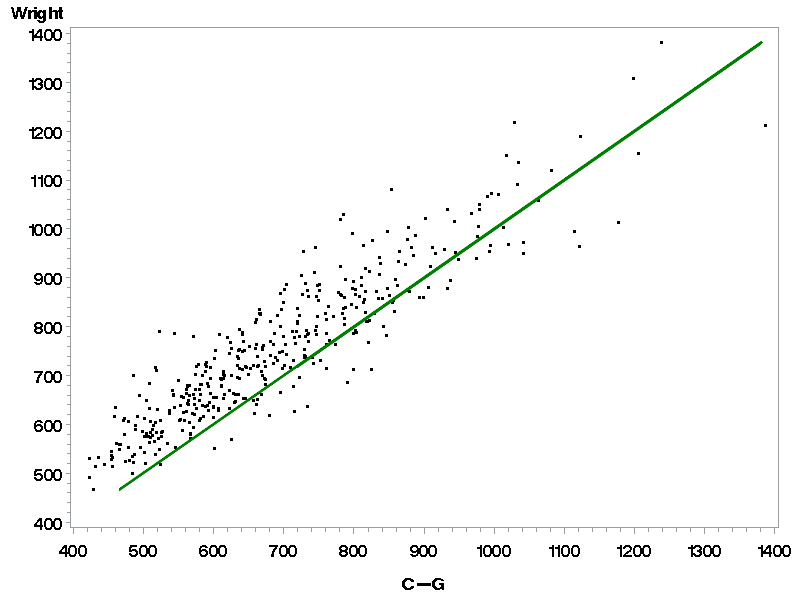

Supplement: Supplementary file 1 [file mmc1.docx]
