# Supplementary material for: Carboplatin versus two doses of cisplatin in combination with gemcitabine in the treatment of advanced non-small-cell lung cancer: Results from a British Thoracic Oncology Group randomised phase III trial
Source: Eur J Cancer. 2017 Sep;83:302–12. doi: 10.1016/j.ejca.2017.05.037 (PMC5597318; doi:10.1016/j.ejca.2017.05.037)
Supplement: Supplementary file 2 [file mmc2.pdf]

# BTOG2

# A British Thoracic Oncology Group

**phase III trial of gemcitabine plus cisplatin at 80mg/m$^2$ versus gemcitabine plus cisplatin at 50mg/m$^2$ versus gemcitabine plus carboplatin AUC 6 in stage IIIB/IV non-small cell lung cancer (NSCLC)**

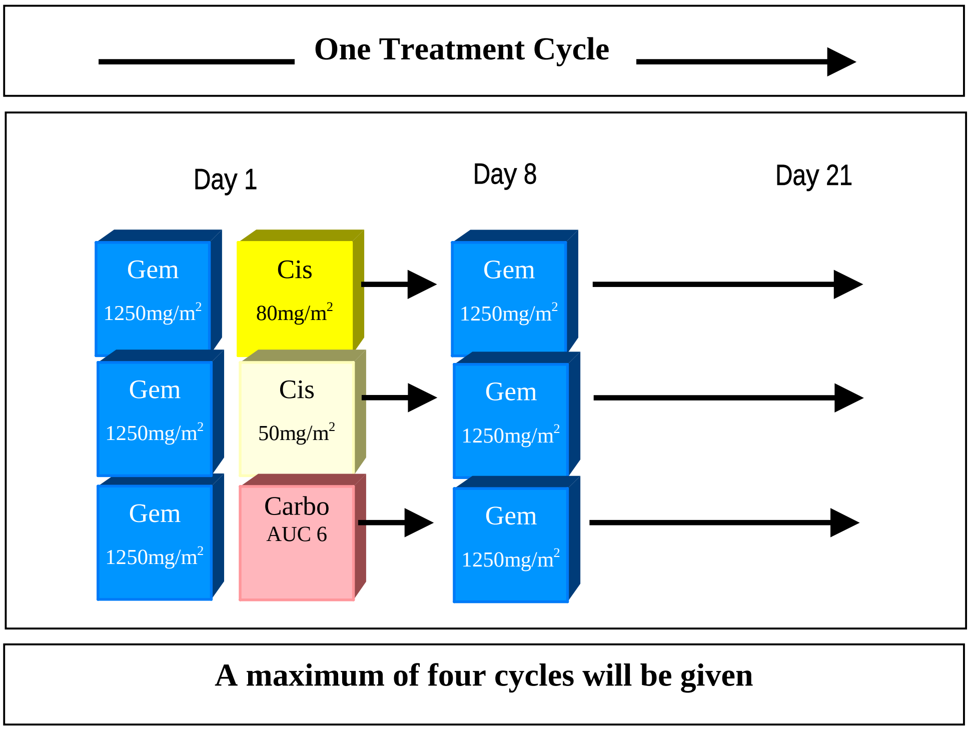


**Version 4.4 dated 06/02/07**

## BTOG2 TRIAL MANAGEMENT GROUP

| | |
|---|---|
| **Prof. David R. Ferry,<br>New Cross Hospital, Wolverhampton** | **Chief Investigator & Clinical Co-ordinator** |
| **Dr Kenneth O'Byrne,<br>St James's Hospital, Ireland** | **Co-investigator & Clinical Co-ordinator** |
| **Dr Lucinda Billingham,<br>Cancer Research UK Clinical Trials Unit,<br>University of Birmingham** | **Co-investigator & Biostatistician** |
| **Professor Philip Johnson,<br>Cancer Research UK Clinical Trials Unit,<br>University of Birmingham** | **Co-investigator & Proteomics Lead** |
| **Dr Rafael Rosell,<br>Hospital Germans Trias i Pujol, Barcelona, Spain** | **Co-investigator & Genomics Lead** |
| **Professor Ian Smith,<br>Royal Marsden Hospital, London** | **Collaborator and Head of the NCRI Lung Cancer Group** |
| **Dr Jenny Barnwell<br>Cancer Research UK Clinical Trials Unit,<br>University of Birmingham** | **Manager for Trial Co-ordination** |
| **Hugh Jarrett<br>Cancer Research UK Clinical Trials Unit,<br>University of Birmingham** | **Trial co-ordination** |

## SPONSOR

**The University of Birmingham**

## TRIAL ADMINISTRATION

**BTOG2 STUDY OFFICE**
**BTOG2 Trial Co-ordinator**
**Cancer Research UK Clinical Trials Unit, Institute for Cancer Studies,**
**The University of Birmingham, Edgbaston, Birmingham B15 2TT**
**Tel: 0121 414 6425 Fax: 0121 414 2230**
**Email:** BTOG2@trials.bham.ac.uk
**Website: www.BTOG2.bham.ac.uk**

## CLINICAL QUERIES

**Clinical queries should be directed to the clinical co-ordinator,**
**Prof David Ferry**
**Tel: 01902 695204.**

## BRITISH THORACIC ONCOLOGY GROUP (BTOG)

**Ms Dawn McKinley British Thoracic Oncology Group Operational Manager**
**Hospital Management Offices, Glenfield Hospital, Leicester LE3 9QP**
**Tel: 0116 250 2811 Fax: 0116 250 2810**

# CONTENTS

***1) INTRODUCTION*** ........ ***7***
*1.1 BACKGROUND* ........ *7*
*1.2 STUDY RATIONALE* ........ *9*
*1.3 PROTEOMIC AND GENOMIC SUB-STUDIES* ........ *9*
***2) STUDY DESIGN*** ........ ***10***
*2.1 TREATMENT ARMS* ........ *10*
*2.2 RECRUITMENT* ........ *10*
*2.3 STUDY OBJECTIVES* ........ *10*
*2.4 ELIGIBILITY CRITERIA* ........ *11*
*2.5 CONCOMITANT MEDICATION* ........ *12*
***3) TREATMENT PLAN*** ........ ***12***
*3.1 DRUG SUPPLIES* ........ *12*
*3.2 ADMINISTRATION OF THE PLATINUM-BASED REGIMENS* ........ *12*
*3.3 SUPPORT TREATMENTS: HYDRATION AND ANTI-EMETICS* ........ *13*
*3.4 DOSE MODIFICATIONS* ........ *14*
*3.5 WITHDRAWAL AND ADDITIONAL TREATMENTS* ........ *15*
***4) STUDY EXAMINATIONS AND ASSESSMENTS*** ........ ***17***
*4.1 PROCEDURES AND CLINICAL ASSESSMENTS* ........ *17*
*4.2 QUALITY OF LIFE ASSESSMENTS* ........ *18*
*4.3 RESOURCE USE ASSESSMENT FOR COSTS* ........ *18*
*4.4 PROTEOMIC AND GENOMIC SUB-STUDIES* ........ *19*
***5) STATISTICAL CONSIDERATIONS*** ........ ***20***
*5.1 STUDY OUTCOME MEASURES* ........ *20*
*5.2 STUDY ANALYSIS* ........ *20*
*5.3 CALCULATION OF SAMPLE SIZE* ........ *21*
*5.4 INDEPENDENT DATA MONITORING COMMITTEE (DMC)* ........ *22*
*5.5 MILESTONES* ........ *22*
***6) ADVERSE EVENT DEFINITION AND REPORTING*** ........ ***22***
*6.1 ADVERSE EVENT DEFINITIONS* ........ *22*
*6.2 ADVERSE EVENT (AE) REPORTING* ........ *23*
*6.3 SERIOUS ADVERSE EVENT (SAE) REPORTING* ........ *24*
*6.4 FOLLOW-UP OF AES* ........ *24*
***7) STUDY ORGANISATION*** ........ ***25***
*7.1 RANDOMISATION PROCEDURE* ........ *25*
*7.2 SITE RESPONSIBILITIES* ........ *25*
*7.3 FORMS AND DATA COLLECTION* ........ *25*
*7.4 PROTOCOL COMPLIANCE AND MONITORING* ........ *25*
*7.5 ARCHIVING* ........ *26*
***8) ETHICAL AND REGULATORY STANDARDS*** ........ ***26***
*8.1 ETHICAL CONDUCT OF THE STUDY* ........ *26*
*8.2 REGULATORY STATUS* ........ *26*
*8.3 PATIENT INFORMED CONSENT* ........ *26*
*8.4 PATIENT CONFIDENTIALITY* ........ *26*
*8.5 PATIENT WITHDRAWAL* ........ *27*
*8.6 PROTOCOL AMENDMENT* ........ *27*
*8.7 SPONSORSHIP AND INDEMNITY* ........ *27*
***9) PUBLICATION POLICY*** ........ ***27***
***10) REFERENCES*** ........ ***28***
***APPENDIX 1) CISPLATIN*** ........ ***31***
***APPENDIX 2) CARBOPLATIN*** ........ ***36***
***APPENDIX 3) GEMCITABINE*** ........ ***41***
***APPENDIX 4) AMERICAN JOINT COMMITTEE ON CANCER STAGING: LUNG CANCER*** ........ ***46***
***APPENDIX 5) WORLD HEALTH ORGANIZATION (WHO) PERFORMANCE STATUS*** ........ ***48***
***APPENDIX 6) FORMULAE*** ........ ***49***
***APPENDIX 7) QUALITY OF LIFE QUESTIONNAIRES*** ........ ***51***
***APPENDIX 8) RECIST CRITERIA*** ........ ***56***
***APPENDIX 9) ABBREVIATIONS*** ........ ***59***

# TRIAL SUMMARY

**Acronym:** BTOG2.
**Title:** **B**ritish **T**horacic **O**ncology **G**roup: - phase III trial of gemcitabine plus cisplatin at 80mg/m$^2$ versus gemcitabine plus cisplatin at 50mg/m$^2$ versus gemcitabine plus carboplatin AUC 6 (Wright[35]) in stage IIIB/IV non-small cell lung cancer (NSCLC)

**Aims** This trial addresses the core issues of the optimal dose of cisplatin in advanced NSCLC, and if carboplatin can safely substitute for cisplatin.

**Outcome Measures:**

**Primary**
- Length of survival

**Secondary**
- Symptom control and quality of life
- Response to treatment
- Dose intensity of chemotherapy
- Ratio of cycles given as in-patient versus out-patient
- Intensity, number and duration of toxic episodes (Grade 2-4)
- Costs and cost effectiveness

**Translational Sub-Studies**

**Proteomic**
- Analysis of proteomic spectra from serum

**Genomic**
- Focused analysis of peripheral blood leucocyte DNA

**Main *(but not exhaustive)* eligibility criteria:**
- Histologically or cytologically confirmed NSCLC (excluding mixed SCLC/NSCLC)
- Radiologically verified stage IIIB (unsuitable for radical radiotherapy) or stage IV disease
- Presence of 1 or more clinically or radiological measurable lesions by RECIST criteria
- Performance status 0, 1 or 2 (WHO performance scale)
- Age ≥18 years
- Life expectancy ≥12 weeks
- Adequate haematological function
- Creatinine clearance: ≥60ml/min (Wright[35])
- Adequate hepatobiliary function
- Able and willing to participate in the quality of life assessment
- Written informed consent

**Main (but not exhaustive) exclusion criteria:**

- Prior chemotherapy or radiotherapy (palliative RT which does not impinge on the lung lesion field is permitted). Prior surgical resection is allowed.
- Evidence of severe or uncontrolled systemic diseases
- Evidence of significant clinical disorder or laboratory finding
- Concomitant or previously malignancy likely to interfere with protocol treatment
- Pre-existing neuropathy grade >2
- Clinically apparent metastatic disease to the brain
- Previous investigational agent in the last 12 weeks
- Male and female patients (of childbearing age) not using adequate contraception

**Diagrammatic representation of treatment allocation:**

Randomise
1350 patients

Arm A
Gem 1250$mg/m^2$ day 1 day 8
Cis 80 $mg/m^2$ day 1
21 day cycle for 4 cycles

Arm B
Gem 1250$mg/m^2$ day 1 day 8
Cis 50 $mg/m^2$ day 1
21 day cycle for 4 cycles

Arm C
Gem 1250$mg/m^2$ day 1 day 8
Carbo AUC 6
(Wright[35])day 1
21 day cycle for 4 cycles

**Investigations required prior to randomisation:**

- Biochemistry to include: Urea, Cr, Na, K, Ca, CK, Alb, Bili, ALP, and AST/ALT within TWO weeks prior to the start of treatment
- Creatinine Clearance: $\geq$60ml/min (Wright[35]) within TWO weeks prior to the start of treatment
- Thoracic CT scan within FOUR weeks prior to the start of treatment
- Chest X-ray within TWO weeks prior to the start of treatment
- Quality of Life Questionnaire

**National trial co-ordinator details:**

BTOG2 Trial Co-ordinator – Hugh Jarrett
Cancer Research UK Clinical Trials Unit, Institute for Cancer Studies,
University of Birmingham, Edgbaston, Birmingham B15 2TT
Tel: 0121 414 6425 Fax: 0121 414 2230 Email: BTOG2@trials.bham.ac.uk
Website: www.BTOG2.bham.ac.uk

# BTOG2 Study

## Schedule of Events

| | **Baseline**[a] | **Cycle 1 Day 1** | **Cycle 1 Day 8** | **Cycle 2-4 Day 1** | **Cycle 2-4 Day 8** | **End of treatment** (Between 4-6 weeks after start of last cycle) | **Follow up** Monthly for 6 months[ψ] |
|---|---|---|---|---|---|---|---|
| History | x | | | | | | |
| Physical examination | x | | | x | | x | x |
| Performance status | x | | | x | | x | x |
| Weight | x | | | x | | x | x |
| Body Surface Area | x | | | x | | | |
| Vital signs | x | | | x | | x | x |
| Full Blood count | x | | | x | | | |
| Biochemistry[b] | x | | | x | | | |
| Creatine Kinase[c] | | x | | x | | | |
| Creatinine Clearance[d] | x | | | x | | | |
| Concomitant meds. | x | x | x | x | x | x | x |
| Thoracic CT scan | x | | | Between day 14 & 21 of cycle 2 only | | Between 3-4 weeks after day 8 chemo. of the final cycle. | |
| Chest X-Ray | x | | | x | | x | x |
| Toxicity | | | x | x | x | x | x |
| Quality of Life | x | | | x | | x | x |

a Baseline: All to be carried out prior to randomisation and within **TWO** weeks prior to the start of treatment, except thoracic CT scan which can be done up to **FOUR** weeks prior to the start of treatment

b Biochemistry to include: Urea, Cr, Na, K, Ca, Alb, Bili, ALP, and AST or ALT

c Creatine Kinase is a key term in the Wright formula

d Creatinine Clearance: $\geq$60ml/min (Wright[35])

ψ Patients will be followed regularly until death or end of trial. Follow-up after 6 months will be as routine, with additional cancer treatments and death to be reported on the appropriate CRF supplied in investigator folder.

# 1) INTRODUCTION

## 1.1 Background

General Information on Non-Small Cell Lung Cancer

Lung cancer is the leading cause of cancer death in men and women in the UK and the rest of the Western world. In the UK there are around 100 lung cancer deaths per day, of which 75-80% are non-small cell lung cancer (NSCLC). 80% of lung cancers are due to cigarette smoking, and for a man of 35 years who smokes more than 25 cigarettes per day the chance of dying of lung cancer before the age of 75 is 13%[1]. Only a small minority of NSCLC cases are potentially treatable with curative intent surgery at diagnosis, ranging from 5%-15% of cases. Thus the vast majority of NSCLC cases are incurable at presentation and candidates for palliative chemotherapy.

Evolution of Cisplatin Based Chemotherapy for NSCLC

Determining the dose of chemotherapy agents to be deployed in the treatment of cancer is an evolving science[2]. Cisplatin introduced in 1967, remains a widely active drug of utility today. The dose of cisplatin used can be limited by emesis, renal damage, neurotoxicity or fatigue[3]. Whilst acute emesis is now much better controlled by 5-HT3 anatgonists[4] and renal damage attenuated by saline hydration[5], no easy answers to fatigue and neurotoxicity have emerged. Despite these problems cisplatin has endured in clinical practice because of its efficacy, especially in germ cell tumours[6], upper gastrointestinal tract cancers[7], bladder cancer[8] and non-small cell lung cancer[9].

Cisplatin was first demonstrated to be an active drug in NSCLC more than 25 years ago[10.] The Medical Research Council (MRC) meta-analysis of 52 randomised trials identified cisplatin as active agent able to prolong life in the neo-adjuvant setting and palliative context in advanced disease. The question of dose has been addressed in a small number of clinical trials[11,12,13,14,15]. The data is far from convincing that increasing the dose from 50mg/m$^2$ three-weekly to 100mg/m$^2$ three-weekly improves outcomes, although one non-randomised trial did report that although response rate was not better for 120mg/m$^2$ versus 60mg/m$^2$, duration of response was doubled to 12 months. More recently a meta analysis of 9074 patients in randomised trials reported that for patients treated with low dose cisplatin of < 60 mg/m$^2$ 3-weekly median survival was 7.2 months versus 9.2 months for patients treated at 78-90 mg/m$^2$ 3-weekly[16]. Not surprisingly there is great heterogeneity in clinical practice with regard to cisplatin dose.

Cisplatin is combined with an increasing number of chemotherapy drugs to treat NSCLC. The first combination regimen to be widely accepted was cisplatin plus vindesine[17], then mitomycin, vinblastine and cisplatin (MVP). The next iteration was to replace the vinca alkaloid with ifosfamide producing the MIC regimen[18]. Literature on this regimen illustrates the heterogeneity of practice well. The first report with MIC utilised a dose of cisplatin of 50mg/m$^2$ three-weekly (MIC50). This dose has subsequently been shown to prolong life and improve quality of life in a randomised trial of 800 patients. Other authors have duplicated the regimen precisely, but others have increased the dose of cisplatin to 100mg/m$^2$ three-weekly (MIC100)[19].

Development of Chemotherapy for NSCLC in the 1990s.

Through the 1990s new drugs emerged as active in combination with cisplatin in NSCLC. They included the vinca navelbine[20], the taxanes, taxotere[21] and taxol[22], the antimetabolite gemcitabine[23, 24] and topisomerase I inhibitors[25]. A sense that these drugs had moved practice forwards with improved response rates and improved median survival became widely accepted with no clear

randomised trial data to support the contention. In addition, variation of cisplatin dose in new combinations is widespread. Also, because taxanes have the potential to cause neuropathy, interest in carboplatin combinations developed, especially taxol/carboplatin, which became the most widely used regimen in the USA.

## Carboplatin in NSCLC

Carboplatin is less neurotoxic and emetic than cisplatin and easy to administer by short infusion but may be more myelosuppressive. The optimal dose of carboplatin in NSCLC has never been systematically addressed. Indeed carboplatin may well be a less active agent than cisplatin in NSCLC[26] as is the case in germ cell, oesophageal cancer and bladder cancer. This issue has been brought into focus by a number of recent studies. In a randomised trial of 422 patients the combination of mitomycin, ifosfamide and cisplatin (50mg/m$^2$) produced a 40% response rate and median survival of 6.5 months, versus a response rate of 37% and median survival of 10.0 months for carboplatin (AUC 5) plus day 1 and 8 gemcitabine[27]. This result is statistically significant, but compared to other randomised trials using MIC, the MIC arm of this trial may have by chance produced a spurious results (see Table 1). In addition a trial of similar design of 372 patients found that the dramatic effect of carboplatin/gemcitabine versus MIC or MVP was not found, with median survival in both arms being 8 months[28]. Furthermore there are two recent large randomised trials that have addressed the issue of cisplatin versus carboplatin in combination with taxol or taxotere[29,30]. Together these trials randomised 1838 patients, and both showed 6-8 week survival advantage for the cisplatin arm, which was at a dose of 75 or 80 mg/m$^2$. Furthermore there was no decrease to quality of life because of receiving cisplatin. Also two small trials of gemcitabine plus cisplatin at 80 mg/m$^2$ 3-weekly versus gemcitabine plus carboplatin both indicate inferior survival for the carboplatin arm[31], the trial of Novakova et al[32] of 176 patients reporting a median survival for the carboplatin arm of 7 months, but the cisplatin arm of 11 months.

## Randomised Trials of Newer Regimens in NSCLC

These concerns have been brought to a head by the data from the randomised trial ECOG1594 that randomised patients with stage IIIB/IV NSCLC to taxol (3hr) plus carboplatin AUC 6, taxol (24hr) plus cisplatin, taxotere plus cisplatin, or gemcitabine plus cisplatin (see Table 1 below)[33]. The first point was the low response rates reported which were much lower than those in the phase II literature. None of the regimens showed a survival advantage over the others, which despite only including performance stage 0/1 patients, was 7.5-8.8 months. These data compared unfavourably with the low cisplatin dose data from the randomised trial of MIC50 versus supportive care, in which patients with PS0/1 did equally as well [18] as those patients treated in ECOG1594.

**Table 1**

| | **ECOG 1594** | | | | **MIC2** |
|---|---|---|---|---|---|
| **Cisplatin dose (mg/m$^2$)** | **75** | **100** | **75** | **AUC 6 carbo** | **50** |
| **Cisplatin dose intensity (mg/m$^2$/week)** | 25 | 25 | 25 | AUC 2 | 17 |
| **Other drug (mg/m$^2$)** | Taxol 135 (24h) | Gemcitabine 1000, d1,d8 | Taxotere 75 | Taxol 225, 3 hr | MMC 6 Ifos 3000 |
| **Response rate (%)** | 21 | 21 | 17 | 15 | 38* |
| **Median survival (mo)** | 7.8 | 8.1 | 7.4 | 8.3 | 8.4* |

*these data relate to the PS0/1 patients in the MIC2 trial

## Adverse reactions

Adverse reactions to cisplatin, carboplatin and gemcitabine are well established and outlined in Appendix 1, 2 and 3 respectively. Also see summary of product characteristics for each study drug.

## 1.2 Study Rationale

In the treatment of advanced NSCLC there is no standard chemotherapy that is clearly superior in terms of survival or quality of life. There is, however, undoubtedly a need to optimise chemotherapy of NSCLC and one step to be taken would be to define the dose of cisplatin that produces the best balance between efficacy and quality of life. Thus if higher dose intensities of cisplatin produce superior survival, as the recent meta analysis on 9074 patients indicates, then it may be worth the toxicity trade off, but if there is no improved survival then optimal dose intensity of cisplatin may be below that which can be delivered. The cisplatin versus carboplatin question has also become a central question needing a clear answer. Carboplatin may be better tolerated and easier to administer than cisplatin but recent large trials in combination with taxanes indicate that carboplatin is less efficacious and there may be no difference in quality of life[29,30]. Indeed, in a recent metanalysis[34] of five trials investigating drug regimens containing platinum plus a new agent, the cisplatin based group yielded an 11% longer survival than the carboplatin based group (p=0.04). Gemcitabine can be easily combined with cisplatin or carboplatin and because of the good toxicity profile and relatively lower cost of gemcitabine compared to taxanes, it is emerging as the leading agent in combination regimens.

We propose conducting a randomised trial of gemcitabine plus either carboplatin or cisplatin at 80mg/m$^2$ or 50mg/m$^2$ in stage IIIB or IV NSCLC. This will define which platinum analogue and which dose of cisplatin in combination with gemcitabine is preferable in this patient group. As quality of life will also be assessed it should be possible to define with confidence these key questions in the management of NSCLC for the first time. In addition any differences in survival and quality of life will be weighed against differences in costs in order to guide health-care decisions in the future. It is anticipated that the trial design will allow all UK cancer centres to enrol patients. Also should newer agents to new targets such as EGFR antagonists become a standard of care they could also be incorporated into regimens used by randomising centres.

## 1.3 Proteomic and Genomic Sub-Studies

To complement the main trial, we aim to recruit as many of the trial patients as possible, into both a proteomic and a genomic sub-study, subject to patient consent and appropriate centre facilities. The main aim of these sub-studies is to explore the possibility of identifying, according to proteomic and genomic factors, patients who are likely to respond to treatment. As approximately 30%, of patients will respond to chemotherapy it would be desirable if we could identify, before the initiation of, or shortly after commencing treatment, those patients who are likely to respond. This would avoid inflicting toxicity on those who fail to respond and could potentially save resources. Studies like these are crucially dependent on the quality of the sample and clinical documentation available and those obtained during a randomised clinical trial are particularly appropriate. Recognising that it is unlikely that significant amounts of fresh tissue will be available in this study, we have focused our scientific sub-studies on serum and blood cell based analysis.

The proteomic sub-study aims to identify an individual serum protein, or more likely combination of serum proteins, that would identify those patients most likely to respond to chemotherapy. Whilst this would be the main aim, the serum sample that we would take could also be used to develop new diagnostic methods and also new methods of assessing response to treatment. This may allow the identification of serum proteins of prognostic significance and is an open-ended approach. The proteomic analysis will be under the direction of Professor Philip Johnson at the Cancer Research

UK Clinical Trials Unit (CRCTU) at the University of Birmingham. This will form part of his on going programme of established research in this area.

The genomic study will be under the direction of Dr Rafael Rosell at the University of Barcelona, Spain who will conduct a focused genomic investigation using peripheral blood leukocyte DNA on known genes that are involved in DNA repair. These genes, ERCC1 and RRM1 may be involved in platinum resistance mechanisms. Clearly it would be useful to profile patients such that those more likely to benefit from treatment could be identified.

Standard Operating Procedures for sub-studies will be supplied.

# 2) STUDY DESIGN

## 2.1 Treatment Arms

This is a multi-centre, three-arm randomised, phase III trial. Patients will be randomised to receive four cycles of one of three different treatment arms as shown in Figure 1.

**Figure 1: BTOG2 Trial Design**

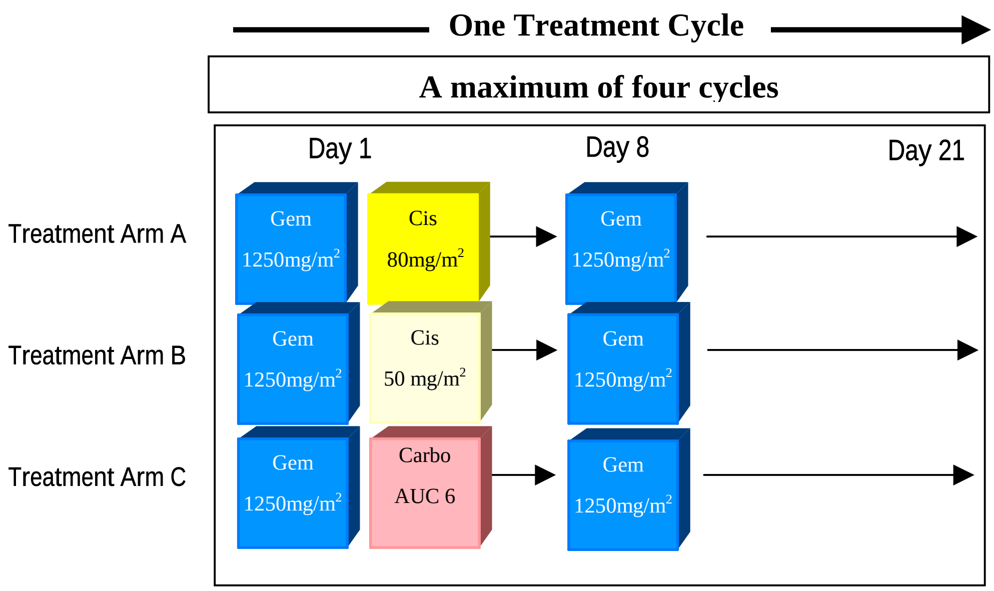


**Gem**, gemcitabine; **Cis**, cisplatin; **Carb**, carboplatin, AUC 6 (Wright[35])

## 2.2 Recruitment

The trial aims to recruit a total of 1350 patients, with 450 patients per treatment arm.

## 2.3 Study Objectives

Primary Objective

To compare the efficacy in terms of survival time of gemcitabine plus cisplatin at 80mg/m$^2$ versus gemcitabine plus cisplatin at 50mg/m$^2$ versus gemcitabine plus carboplatin AUC 6 (Wright[35]), in patients with advanced NSCLC.

Secondary Objectives

To compare the efficacy of gemcitabine plus cisplatin at 80mg/m$^2$ versus gemcitabine plus cisplatin at 50mg/m$^2$ versus gemcitabine plus carboplatin AUC 6 (Wright[35]) in terms of:

1) Symptom control and quality of life
2) Response to treatment
3) Dose intensity of chemotherapy
4) Ratio of cycles given as in-patient versus out-patient
5) Intensity, number and duration of toxic episodes (Grades 2-4)
6) Costs and cost-effectiveness

In addition two sub-studies aim to complement the main trial. The main aim of these sub-studies is to explore the possibility of identifying, according to proteomic and genomic factors, patients who are likely to respond to treatment.

## 2.4 Eligibility Criteria

Inclusion Criteria

- Histologically or cytologically confirmed NSCLC (excluding mixed SCLC/NSCLC)
- Radiologically verified stage IIIB (unsuitable for radical radiotherapy) or stage IV disease (see Appendix 4)
- Presence of 1 or more clinically or radiological measurable lesions by RECIST criteria (see Appendix 8)
- Performance status 0, 1 or 2 (WHO performance scale – see Appendix 5)
- Age $\geq$18 years
- Life expectancy $\geq$12 weeks
- Adequate haematological function: haemoglobin ≥10g/dl; WBC ≥3.0 x $10^9$/L; absolute neutrophil count (ANC) ≥1.5 x $10^9$/L; platelet count ≥100,000/mm$^3$
- Creatinine clearance: $\geq$60ml/min (Wright[35], see Appendix 6)
- Hepatobiliary function: Bilirubin <1.5xULN, Alkaline phosphatase (ALP) <2xULN, AST/ALT <3.0xULN or <5xULN in the presence of liver metastases
- Patient compliance and geographic proximity that allows adequate follow-up
- Able and willing to participate in the quality of life assessment
- Written informed consent

Exclusion Criteria

- Prior chemotherapy or radiotherapy (palliative RT which does not impinge on the lung lesion field is permitted), however prior surgical resection is allowed provided no neo-adjuvant or adjuvant chemotherapy was given
- Evidence of severe or uncontrolled systemic diseases that in the view of the investigator, makes it undesirable for the patient to participate in the trial
- Evidence of significant clinical disorder or laboratory finding which, in the opinion of the investigator makes it undesirable for the patient to participate in the trial
- Concomitant or previously malignancy likely to interfere with protocol treatment or comparisons
- Pre-existing neuropathy grade >2

- Clinically apparent metastatic disease to the brain
- Unresolved toxicity or incomplete recovery from previous surgery
- Psychiatric disorder making reliable informed consent impossible or that might prevent completion of treatment or follow-up
- Previous investigational agent in the last 12 weeks
- Male and female patients (of childbearing age) not using adequate contraception
- Female patients who are pregnant or breast-feeding

### 2.5 Concomitant Medication

No other cytotoxic chemotherapy, immunotherapy, hormonal therapy (excluding contraceptives and replacement steroids) or experimental medications will be permitted while patients are on the study. Any disease progression requiring other forms of specific anti-tumour therapy will be a cause for early discontinuation of protocol treatment. Patients should receive full supportive care. Patients may receive growth factors for prolonged myelosuppression.

## 3) TREATMENT PLAN

### 3.1 Drug Supplies

Prescribing should be initiated by the investigator and a continued supply of drugs arranged according to normal local practice. See Appendices 1, 2 and 3 for more information on cisplatin, carboplatin and gemcitabine respectively. Appropriate labelling requirements should be carried out in pharmacy. Labels will be supplied by the BTOG2 Study Office, but a Centre may use their own labels provided they are submitted to, and approved by, the BTOG2 Study Office.

### 3.2 Administration of the Platinum-Based Regimens

1) Dose and schedule for administration of the platinum-based regimens is given in Tables 2 & 3
2) Treatment must start within TWO weeks of all baseline assessments, with the exception of the thoracic CT, in which case regimens can be administered within FOUR weeks
3) Treatment can be administered in the out-patient or in-patient setting, but it is anticipated most cycles will be delivered as an out-patient episode
4) Administration of carboplatin must be according to creatinine clearance as determined by the Wright[35] calculation (see Appendix 6), with the AUC constant of 6 being used to determine the carboplatin dose.
5) Carboplatin doses are to be calculated via “GFR calculators” supplied by the BTOG2 Study Office (as spreadsheets in an Excel format and as a printed sheet to be completed by hand). Whilst these calculators are available for both Jaffe and enzymatic methods of serum creatinine measurement, centres should notify the study office which method is to be used, so the appropriate calculator can be supplied. Examples of the GFR calculators are supplied in Appendix 6.
6) If the dose of carboplatin would change by more than 5% from one cycle of treatment to the next, then the dose should be altered. For changes less than this, the dose remains the same.

### 3.3 Support Treatments: Hydration and Anti-Emetics

The following support treatments are strongly recommended for the trial treatment regimens, and there should be a sound pharmacological basis to any local variations if they are to be acceptable. **Prior consultation with the BTOG2 Study Office should be sort before any local regimens are implemented.**

Cisplatin-Based Treatments
Treatment Arm A: gemcitabine plus cisplatin 80mg/m$^2$
Treatment Arm B: gemcitabine plus cisplatin 50mg/m$^2$

**Table 2 Cisplatin-Based Regimens Plus Support Treatments**

| | Time (hours) | Drug | Fluid |
|---|---|---|---|
| **DAY 1** | 0 | Bolus 5-HT$_3$ antagonist | - |
| | | Dexamethasone 8mg iv | - |
| | 0 – 2 | | N saline 1 litre |
| | 2 – 2.5 | Gemcitabine 1250mg/m$^2$ | N saline 0.25 litre |
| | 2.5-3 | Mannitol 20% solution iv | 0.2 litre |
| | 3-4 | Cisplatin 50 or 80mg/m$^2$ | N saline 0.5 litre |
| | 4-6 | 20 mmol KCl + 1g $MgSO_4$ | N saline 1 litre |
| **DAY 8** | 0<br>0 – 0.5 | Dexamethasone 4mg iv<br>Gemcitabine 1250mg/m$^2$ | -<br>N saline 0.25 litre |

The use of mannitol will ensure good diuresis and although urine output can be measured at investigator's discretion it is not considered necessary. Standard anti-emetics should be 5 days of a 5-HT3 antagonist plus dexamethasone 4mg twice daily. After day 8 gemcitabine, most patients will need only oral domperidone 20mg up to 4 times daily as required. If patients have acute uncontrolled emesis then consideration to admission for intravenous fluid support should be given.

Carboplatin-Based Treatment
Treatment Arm C: gemcitabine plus carboplatin AUC 6 (Wright[35])

**Table 3 Carboplatin-Based Treatment Plus Support Treatments**

| | Time (hours) | Drug | Fluid |
|---|---|---|---|
| **DAY 1** | 0 | Bolus 5-HT$_3$ antagonist & Dexamethasone 8mg iv | - |
| | 0-0.5 | Gemcitabine 1250mg/m$^2$ | N saline 0.25 litre |
| | 0.5-1.5 | Carboplatin AUC 6 (Wright[35]) | 5 % dextrose 0.5 litre |
| **DAY 8** | 0 | Dexamethasone 4mg iv | |
| | 0-0.5 | Gemcitabine 1250mg/m$^2$ | N saline 0.25 litre |

Standard anti-emetics for day 1 should be 5 days of a 5-HT$_3$ antagonist or domperidone plus dexamethasone 4mg twice daily. After day 8 gemcitabine, most patients will need only oral domperidone 20mg up to 4 times daily as required.

## 3.4 Dose Modifications

**There will be no dose escalations but there will be dose reductions due to haematological and non-haematological toxicity and neurotoxicity. In addition there will be dose reductions for cisplatin-induced renal toxicity and ototoxicity**

Dose Reductions for Haematological Toxicity

**Table 4 Dose Reductions for Haematological Toxicity**

| **ANC x 10$^9$/L** | | **Platelets x mm$^3$** | **Day 1- gemcitabine and either cisplatin/carboplatin Day 8 - gemcitabine** |
|---|---|---|---|
| > 1.5 | and | ≥ 100,000 | 100% of both drugs |
| 0.5 – 1.5 | or | 50,000 to 99,000 | 75% of day 1 both drugs* and day 8 gemcitabine* |
| < 0.5 | or | <50,000 | **Delay$^{\dagger}$ $^{Day\ 1}$ or Omit $^{Day\ 8}$** |

dose reduction is maintained into subsequent cycles

† If delay is >3 weeks the patient will be withdrawn from protocol treatment

If toxicity occurs on day 1 of cycle then the patient should be reassessed weekly. For patients whose ANC drops below 0.5 x 10$^9$/L or platelets of < 50,000 a delay should occur until recovery to the 100% of dosage criteria are met. **The patient will then receive all subsequent doses at the 75% level**. In the event of further haematological toxicities as above, the dose will be further reduced to 75% of this already reduced dose.

Dose Reduction for Non-Haematological Toxicity

**Table 5 Dose Reductions for Non-Haematological Toxicity**

| **NCI CTCAE v 3.0 Grade** | **Action** |
|---|---|
| ≤ 2<br>(Except for nausea/vomiting and alopecia) | 100%** of both drugs |
| ≥ 3<br>(Except for nausea/vomiting and alopecia)<br>(see below for neurotoxicity) | Delay* until recovery to baseline, then resume treatment at a reduced dose level deemed appropriate by the principal investigator. |

* If delay is >3 weeks the patient will be withdrawn from protocol treatment

** Investigator discretion to whether a particular non-haematological toxicity requires dose reduction or treatment delay.

Dose Reductions for Neurotoxicity

**Table 6 Dose Reductions for Neurotoxicity**

| NCI CTCAE Grade | Platinum Dose | Gemcitabine Dose |
|---|---|---|
| 0-1 | 100% | 100% |
| 2 | 50%* | 100% |
| 3 | Omit | 100% |
| 4 | Discontinue patient | |

*Doses should remain reduced in subsequent cycles.

Dose Reductions for Cisplatin-Induced Renal Toxicity

If serum creatinine is raised on day 1 of any chemotherapy cycle then the investigator should check measured clearance and reduce dose as indicated in Table 7.

**Table 7 Dose Reductions for Cisplatin-Induced Renal Toxicity**

| Creatinine clearance (Wright[35]) | Percent of Full Dose |
|---|---|
| >60ml/min | 100% |
| 40-59ml/min | 50% |
| <40ml/min | omit |

Dose Reductions for Cisplatin Induced Ototoxicity

In case of tinnitus or significant clinical hearing loss, cisplatin therapy should be reduced or stopped. The clinician will make the decision regarding cisplatin dosing and also whether to continue gemcitabine alone or to withdraw the patient.

Dose Reductions for ALL Toxicities

If treatment suspended for >3 weeks the patient will be withdrawn from protocol treatment (Section 3.5, below)

Dose reductions related to changes in patient weight

If, on cycles 2-4, the patient's weight changes such that using the cycle 1 dose in mgs would result in a change of more than 5% of the dose in mg/m$^2$, then the mg dose should change accordingly. For weight changes that give less than this variation, no changes in dose are necessary. There is no dose capping required for BSAs in excess of 2.

## 3.5 Withdrawal and Additional Treatments

Termination of Treatment

Below are the criteria for early termination of protocol treatment.

The treatment will stop prematurely in the following cases:

- Intolerable side effects as judged by the investigator or patient
- Treatment is suspended for >3 weeks
- Patient decision to discontinue treatment
- Pregnancy
- Grade 3 (except vomiting) or Grade 4 non-haematological toxicity or symptomatic Grade 4 haematological toxicity despite dose modification

- Tumour progression or stable disease, without any obvious symptomatic improvement, following protocol mandated CT scan on completion of cycle 2
- Serious systemic allergic response to any of the study drugs, e.g. angio-oedema, anaphylaxis or bronchoconstriction
- Withdrawal is considered appropriate for any other reason by the investigator

At treatment withdrawal, a full assessment will be performed consisting of a clinical examination (including vital signs, weight, and WHO Performance status score), FBC and biochemistry profile, estimation of creatinine clearance, quality of life and (Grade 2-4) adverse event monitoring (until resolution of all toxicities).

**If not already carried out, patients coming off study early due to progressive disease or other reasons should whenever possible have disease measured by CT scanning.**

*Despite early termination of protocol treatment, all patients will continue to be followed-up for measurement of all outcomes.*

## Additional Treatment

Those patients who have terminated protocol treatment can receive additional treatment with:

*a) Radiotherapy*

Palliative setting - there should be an interval of 7 days between the last dose of gemcitabine and the start of palliative radiotherapy.
Radical - there should be an interval of 14 days between the last dose of gemcitabine and the start of radical radiotherapy.
Concurrent chemo-radiotherapy with gemcitabine is still experimental and should not be used in patients who have terminated protocol treatment.

*b) Chemotherapy*

Patients can have any form of second-line chemotherapy
EXCEPT cisplatin if they were in the carboplatin arm or carboplatin if in a cisplatin arm.

# 4) STUDY EXAMINATIONS AND ASSESSMENTS

## 4.1 Procedures and Clinical Assessments

Patient monitoring, blood tests, clinical and radiological assessments will be conducted as defined in the chart below. All clinical assessments are in accordance with standard clinical practice.

| | Base-line[a] | Cycle 1 Day 1 | Cycle 1 Day 8 | Cycle 2-4 Day 1 | Cycle 2-4 Day 8 | End of treatment (Between 4-6 weeks after start of last cycle) | Follow up Monthly for 6 months[ψ] |
|---|---|---|---|---|---|---|---|
| History | x | | | | | | |
| Physical examination | x | | | x | | x | x |
| Performance status | x | | | x | | x | x |
| Weight | x | | | x | | x | x |
| Body Surface Area | x | | | x | | | |
| Vital signs | x | | | x | | x | x |
| Full Blood count | x | | | x | | | |
| Biochemistry[b] | x | | | x | | | |
| Creatine Kinase[c] | | x | | x | | | |
| Creatinine Clearance[d] | x | | | x | | | |
| Concomitant medication | x | x | x | x | x | x | x |
| Thoracic CT scan | x | | | x* | | x** | |
| Chest X-Ray | x | | | x | | x | x |
| Toxicity | | | x | x | x | x | x |
| Quality of Life | x | | | x | | x | x |

a Baseline: All to be carried out prior to randomisation and within **TWO** weeks prior to the start of treatment, except thoracic CT scan which can be done up to **FOUR** weeks prior to the start of treatment

b Biochemistry to include: Urea, Cr, Na, K, Ca, Alb, Bili, ALP, and AST/ALT

c Creatine Kinase is an integral part of the Wright equation

d Creatinine Clearance: $\geq$60ml/min (Wright[35])

ψ Patients will be followed regularly until death or end of trial. Follow-up after 6 months will be as routine, with additional cancer treatments and death to be reported on the appropriate CRF supplied in investigator folder.

* **CT scanning of tumour:** should be carried out between day 14 & 21 of cycle 2 to avoid treatment delay and subsequent decrease in dose intensity. CT scans are not required during cycle 3 and 4.

** **CT scanning of tumour:** Carried out between 3-4 weeks after the administration of the day 8 gemcitabine of the final cycle.

*NOTE*

After 2 cycles of chemotherapy patients will have a CT scan to define response according to RECIST criteria (see Appendix 8) and the following action will be taken:

(1) Those patients with progressive disease will discontinue randomised treatment

(2) Those patients with response will be allowed to continue randomised treatment if they wish

(3) Those patients with stable disease and without obvious symptom improvement should discontinue randomised treatment

(4) Those with stable disease and with symptom improvement may be offered the opportunity of continuing randomised treatment.

### 4.2 Quality of Life Assessments

Quality of life is an important outcome measure in this study and *therefore all patients are required to participate in this aspect of the trial*. A named person at each participating centre must be nominated to take responsibility for the administration, collection and checking of quality of life questionnaires. Procedures for the quality of life assessments and guidelines for ensuring optimal compliance will be supplied by the BTOG2 Study Office.

Quality of life will be assessed using the EORTC QLQ-C30 and LC13 together with the EuroQol EQ-5D questionnaires (see Appendix 7). The EORTC questionnaires will assess the general and lung cancer specific aspects of quality of life whilst the EuroQoL questionnaire will enable the patient's current health state to be valued for use in calculating quality-adjusted life years and cost-effectiveness.

The timing of assessments will be coordinated with routine clinic visits. For all patients, quality of life will be assessed from pre-treatment up to 6 months post-treatment at the following times:

- Baseline taken prior to day 1 of cycle 1 and *prior to being informed of treatment allocation*
- Day 1 of cycle (2-4) of treatment received, prior to receiving treatment on that cycle
- End of treatment visit (i.e first clinic visit post-treatment) or withdrawal from protocol treatment
- Each monthly follow-up visit post-treatment, up to 6 months from end of treatment.

Patients should complete the questionnaires prior to treatment whilst waiting to be seen in clinic, ideally in a quiet area and without conferring with friends or relatives. Questionnaires will be collected before the patient leaves at which time they will be checked for any missing responses and patients will be asked to complete any missing items. The importance of completing quality of life questionnaires should be emphasised to patients and reasons for non-compliance will be recorded.

### 4.3 Resource Use Assessment for Costs

The treatment arms are expected to differ in cost because of differential drug costs and practices in delivery of treatment. Data will be collected at each clinic visit up to 6 months from end of treatment on key aspects of patient care as follows:

- duration of in-patient episodes
- number of out-patient visits
- doses of trial drugs received
- any additional treatment given following termination of protocol treatment

This resource usage will be multiplied by appropriate unit costs and summed to give a total cost per patient.

## 4.4 Proteomic and Genomic Sub-Studies

Proteomic Sub-study

Subject to patient consent and appropriate centre facilities for collection and storage of whole blood, as many trial patients as possible will have four 5 ml samples of whole blood drawn; the first immediately prior to administration of the first cycle of therapy, the second immediately after the infusion of the study drugs and the third at the end of the day 1 hydration regimen. The final sample will be collected on day 8 of cycle 1 immediately prior to the protocol mandated treatment. The blood should be spun down and serum separated within 2 hours of being taken and stored at -80$^o$C until dispatch to the Institute for Cancer Studies at the University of Birmingham, for analysis. Proteomic techniques, particularly protein chip and ICAT^ technology, will be used to discover so-called, "protein signatures". Such tests could be used alone, or in conjunction with currently available clinical indicators, or with other molecular tests.

The aim of the proteomic analysis, as described in 1.3, is to identify a serum protein or more likely combination of serum proteins that would characterise those patients most likely to respond to chemotherapy. Sampling at baseline, during day 1 and at day 8 of cycle 1 enables either the baseline protein signature or change in signature during and after day 1 of chemotherapy to be related to response. The initial proteomic analysis will be undertaken once the first 500 pairs of serum have been collected. This should ensure with a 30% response rate, that there are at least 150 'responders' and an appropriate number of 'non-responders' to act as control subjects. If the initial analysis suggests that it is indeed possible to predict response on the basis of the initial, or first post-treatment sample, samples from remaining trial patients will be collected for a blinded study to validate the initial results in a prospective manner.

The Clinical Proteomics Group at the Institute for Cancer Studies has all the facilities to carry out this project. This includes SELDI equipment and 2 ion traps which can be used to determined the nature of the proteins contained within the relevant signatures. The group also has a track record in this novel approach to the detection of serum markers for diagnosis and assessment of response.

Genomic Sub-study

Subject to patient consent and appropriate centre facilities, venous blood (2 tubes with 10 ml) will be collected at baseline from each patient into tubes containing EDTA/K3 (Becton Dickinson Reference Number 368457). These samples will be stored at the centre and shipped at regular intervals to Dr Rafael Rosell’s laboratory at University of Barcelona, Spain.

Rosell’s group will isolate genomic DNA from peripheral blood leukocytes after standard erythrocyte lyses with the QIAmp® DNA blood Mini kit (Qiagen, Germany), according to manufacturer's instructions. SNPs in genes of interest will be assessed using the 5' nuclease allelic discrimination assay (Taqman) in a ABI Prism 7000 or 7900HT Sequence Detection System (Applied Biosystems, Foster City, CA, USA).

The aim of the focused genomic analysis will be to assess if the levels of expression of ERCC1 and RRM1 are involved with platinum resistance mechanisms. This analysis would enable the profiling of patients such that those more likely to benefit from treatment could be identified.

Standard Operating Procedures for sub-studies will be supplied.

# 5) STATISTICAL CONSIDERATIONS

## 5.1 Study Outcome Measures

Primary Outcome Measure

- **Length of survival:** defined in whole days, as the time from randomisation into the trial to death from any cause; for those patients who are not observed to die during the course of the study, the length of survival will be censored at the last follow-up date.

.

Secondary Outcome Measures

- **Symptom control and quality of life:** measured using the EORTC QLQ-C30 and LC13 instruments together with EuroQol EQ-5D (see Appendix 7).
- **Response to treatment:** measured using RECIST criteria (see Appendix 8).
- **Dose intensity of chemotherapy:** as each treatment cycle is planned to occur over 3 weeks, one cycle delivered at full dose will be defined as 100%. Treatment delay and day 8 gemcitabine reductions will be taken into account.
- **Ratio of cycles given as in-patient versus out-patient:** to be recorded in whole days, on the Clinical Report Forms (CRFs).
- **Intensity, number and duration of toxic episodes (Grade 2-4):** intensity assessed using the NCI CTCAEversion 3.0 (see Investigator Folder)
- **Costs and cost effectiveness:** the number and duration of in-patient episodes (in whole days), the number of out-patient visits, the doses of trial drugs and any additional treatment given following termination of protocol treatment will be recorded on the CRFs; this resource usage will be multiplied by appropriate unit costs and summed to give a total cost per patient. The difference in costs will be weighed against the differences in survival and quality of life in order to assess the cost effectiveness.

## 5.2 Study Analysis

Analysis of Outcome Measures

The primary research question to be addressed is to compare the efficacy in terms of survival time of gemcitabine plus cisplatin at 80mg/m$^2$, gemcitabine plus cisplatin at 50mg/m$^2$ and gemcitabine plus carboplatin AUC 6 (all regimens being given 3-weekly) in patients with advanced NSCLC. The evidence from previous studies is inconsistent in terms of which treatment arm is expected to be superior and each may be considered 'standard practice', therefore the trial will treat all treatment arms equivalently and aims to investigate any difference between any of the arms.

The primary aim is to test the null hypothesis of no difference between the three treatment arms. The survival for the three treatment arms will be compared in one analysis on an intention to treat basis, using Kaplan-Meier survival curves and a log-rank test. Models for survival data that account for other prognostic factors as well as treatment will also be considered. If a statistically significant difference is found between the three treatment groups at the 5% level then further investigation to determine which pairs of treatment arms differ will be carried out, with tests adjusted to account for the overall type I error.

Since there may be potential gains in quality of life with the lower cisplatin dose or with carboplatin compared to cisplatin, it is important that the analysis assesses non-inferiority in terms of survival when no significant difference between treatment arms is found in the primary analysis. Thus, in addition to the primary analysis, it may be appropriate in the certain circumstances to consider a secondary analysis assessing non-inferiority. In a three-arm trial there are a number of outcome scenarios: (i) clearly if one treatment arm is superior to the other two arms then no further analysis is required; (ii) if one arm is inferior to the other two then the question of non-inferiority between the two superior arms needs to be addressed; (iii) in the event that no treatment arm is obviously superior then the secondary analysis would consist of assessing non-inferiority between each pair of arms. In this third scenario, if non-inferiority is established between the two cisplatin arms then these two arms would be combined to compare with carboplatin in assessment of non-inferiority. It is widely accepted by the lung cancer community that a difference in median survival of 6 weeks would be the largest difference between treatments that can be judged as clinically acceptable. A non-inferiority margin of 6 weeks is therefore chosen as relevant for this trial. Statistical analysis will be based on one-sided 95% confidence intervals. Non-inferiority will be inferred when the entire confidence interval falls above the non-inferiority margin.

Quality of life data will be analysed using longitudinal statistical methods and consideration will be given to missing data that occurs due to dropout and death. The balance between quality of life and survival will be analysed by comparing treatments in a quality-adjusted survival analysis. Response will be analysed according to RECIST guidelines (see Appendix 8 *Section 5*) and response rates will be compared using chi-square tests. Dose intensity and ratio of cycles given in the in-patient versus out-patient setting will be compared using either t-tests or Wilcoxon tests depending on the distribution of the data. Toxicity data will be reported descriptively.

Mean difference in cost between treatment arms and the associated 95% confidence interval will be estimated using non-parametric bootstrapping to account for the skewed distribution of the cost data. Survival and utility measures obtained from the EQ-5D will be combined to give quality-adjusted life years (QALYs). Efficiency issues will be explored using an incremental cost-effectiveness analysis, where the additional cost per QALY gained will be calculated.

Subgroup Analyses

Treatment efficacy will be investigated descriptively for subgroups of patients defined by performance status.

Interim Analysis

Interim analyses will be carried out annually and presented to an Independent Data Monitoring Committee (DMC). The interim analyses will present recruitment data and data on all primary and secondary outcome measures and will include a Bayesian analysis.

Final Analysis

The study is expected to complete recruitment within 3 years. Final analysis will be carried out after all patients have been followed up for at least one year.

### 5.3 Calculation of Sample Size

Sample size calculations are based on the primary outcome of survival time. As described in 5.2 the primary analysis is to compare all three arms against each other in a single analysis in order to determine if there are any differences between the treatments. A log-rank test (two-tailed and with a

5% significance level) will be used to compare the survival of the three treatment arms in a *single* analysis. For a three-arm comparison, 400 *deaths* are required per arm to enable a difference in median survival of two months (7 versus 9 months) to be detected between any of the three arms with 90% power. This is equivalent to a difference in 1-year survival rates of the order of 35% versus 45%. Assuming an accrual period of 3 years and a follow-up period of 1 year, 450 *patients* per arm would need to be recruited to the trial to achieve the required number of events.

As described in 5.2 a secondary analysis, if appropriate, will be carried out to assess non-inferiority between treatment arms. It is widely accepted by the lung cancer community that a difference in median survival of 6 weeks would be the largest difference between treatments that can be judged as clinically acceptable. A non-inferiority margin of 6 weeks is therefore chosen as relevant for this trial. With 400 deaths per arm and using a one-sided 95% confidence interval there will be 80% power to detect non-inferiority of 6 weeks or less (Based on methodology described by Walter Gregory in Appendix 8 of the London Lung Cancer Group Study 11 Protocol)

### 5.4 INDEPENDENT DATA MONITORING COMMITTEE (DMC)

The data will be supplied to an independent DMC, which will be asked to give advice on whether the accumulated data from the trial, together with the results from other relevant trials, justifies the continuing recruitment of further patients. The committee will meet one year after the trial opens and then annually thereafter until the trial closes to recruitment. The DMC may consider discontinuing the trial if the recruitment rate or data quality are unacceptable or if there are cases of excessive toxicity. The trial would also stop early if the interim analyses showed differences between treatments that were deemed to be convincing to the clinical community.

### 5.5 Milestones

The study aims to complete recruitment of 1350 patients within 3 years. We expect a recruitment rate of between 20-25 per month in the first year, rising to 45 per month in the second and third years. The trial will open to recruitment in March 2005 with expected completion of recruitment by March 2008. The anticipated schedule is as follows:

| | |
|---|---|
| March 2005 | Open trial to recruitment |
| October 2006 | 270 patients recruited; 1st report to DMC |
| October 2007 | 700 patients recruited; 2nd report to DMC |
| September 2009 | 1350 patient recruited; close trial to recruitment |
| June 2010 | Final analysis |

## 6) ADVERSE EVENT DEFINITION AND REPORTING

### 6.1 Adverse Event Definitions

<u>Adverse Event</u>

An adverse event is defined as any untoward medical occurrence in a subject to whom a drug has been administered; the event does not need to have a causal relationship to the study drug(s), but symptoms of the targeted cancer should not be classed as an adverse event.

<u>Serious Adverse Event (SAE)</u>
A serious adverse event is any untoward medical occurrence that at any dose:

- Results in death
- Is life-threatening*
- Requires in-patient hospitalisation or prolongation of existing hospitalisation**
- Results in persistent or significant disability/incapacity
- Is a congenital anomaly/birth defect
- Is a new primary cancer
- Is the result of an overdose (whether accidental or intentional)

Other important medical events which neither result in death, nor are life-threatening, nor require hospitalisation, may be considered serious and adverse if judged to jeopardise the patient and require medical or surgical intervention to prevent one of the outcomes listed above (excluding cancer or result of overdose).

*The term 'life-threatening' in the definition of 'serious' refers to an event in which patient was at risk of death at time of the event; it does not refer to an event that hypothetically might have caused death if it were more severe.

**Hospitalisation is defined as an in-patient admission, regardless of length of stay, even if the hospitalisation is a precautionary measure, for continued observation. Hospitalisation for pre-existing condition, including elective procedures, which has not worsened, does not constitute a serious adverse event.

<u>Unexpected Event</u>
This is an adverse event that is not listed as a known toxicity of the study drug (see Appendix 1, 2, 3).

## 6.2 Adverse Event (AE) Reporting

Toxicities will be reviewed using the National Cancer Institute Common Terminology Criteria for Adverse Events (CTCAE) version 3, a copy of which will be enclosed in the Investigator Folder. Any toxicity incurred but not categorised by the NCI CTCAE version 3 should be graded by the physician and be recorded using a scale of (1) mild, (2) moderate, or (3) severe on the CRF.

Only <u>Grade 2-4</u> AEs <u>believed to be related to the study drugs</u> will be recorded on the CRFs. **All new AEs will be recorded up to 30 days after the last treatment cycle on study or until the start of other anti-cancer treatment, whichever occurs first**.

A pre-existing condition should not be reported as an adverse event unless the condition worsens or episodes increase in frequency during the adverse event-reporting period.

## 6.3 Serious Adverse Event (SAE) Reporting

In the case of serious adverse event, whether it is thought to be related or not to study treatment, the investigator must **immediately (within 24hrs)**:

- **Complete** an SAE Form
- **Send** (by fax <u>within 24 hrs</u> of becoming aware of the event) the signed and dated 'Serious Adverse Event Form' to the **BTOG2 Study Office**:

**Fax (24 Hours): 0121 414 2230**

**A receipt of the SAE form will be either be faxed or e-mailed back to the centre within 2 working days. Please telephone the BTOG2 Study Office if a response is not received within this period.**

- **Telephone** (on day of awareness) the **BTOG2 Study Office** at the CRCTU, Birmingham in the case of death or life-threatening events:

**☎: 0121 414 6425**

- 
- **NOTIFY** the NHS Trust as determined by local policy (there is no requirement to report SAEs to local ethics committee (LREC) of the event unless required by local policy

It is the responsibility of the local investigator to assess seriousness, relatedness and expectedness when reporting an SAE. The Chief Investigator (or Deputy) will also independently determine the seriousness, causality and expectedness of the event. It is the responsibility of the Chief Investigator or designee to report suspected unexpected serious adverse reaction (SUSAR) or a suspected serious adverse reaction (SSAR), as determined by the local investigator or Chief Investigator, to the relevant regulatory authorities e.g. Medicines and Healthcare products Regulatory Agency (MHRA) according to UK requirements and the Multi-Centre Research Ethics Committee (MREC).

The **BTOG2 Study Office** will send a safety report to MREC annually and send a copy to all persons in accordance with the EU Directive 2001/20/EC. Principal Investigators are not required to forward this report to their local ethics committee unless specifically requested by their LREC.

<u>Exceptions to Expedient Reporting</u>

Death due to NSCLC does not need to be reported as an SAE, but patient death should be reported on the appropriate CRF supplied in the Investigator Folder.

## 6.4 Follow-Up of AES

Grade 2-4 AEs deemed by the local investigator to be possibly related to the trial medication will be followed until resolution or initiation of other anti-cancer therapy, whichever occurs first. Follow-up information on ongoing AEs of Grade 2-4 will be noted on the relevant CRF and marked as "ongoing".

In the case of an SAE the follow-up should be until resolution (or the investigator assesses it to be chronic or stable) or initiation of other anti-cancer therapy, whichever occurs first. Follow-up should

be noted on the "SAE Form" and be sent to the **BTOG2 Study Office** as information becomes available. Extra annotated information and/or copies of test results may be provided separately.

# 7) STUDY ORGANISATION

**BTOG2** will be co-ordinated for the **BTOG2 Steering Committee** by the CRCTU in Birmingham.

## 7.1 Randomisation Procedure

Randomisation will take place after patient consent has been gained and within **TWO** weeks of all baseline assessments except thoracic CT which can be done up to **FOUR** weeks prior. An eligibility form should be completed prior to randomisation. These details can be phoned or faxed through to the **BTOG2 Study Office** at the CRCTU, Birmingham.

**☎:0800 371 969 or 0800 731 7625; 9am-5pm Monday to Friday.**

**Fax: 0800 328 6412**

After checking eligibility and recording baseline patient details, treatment will be allocated by computer using a minimisation algorithm. Randomisation will be stratified by performance status (0, 1 and 2), stage (IIIB and IV) and centre to ensure that there is a balance between treatments within the strata defined by these key prognostic factors. The allocated treatment and trial number will be given over the telephone and confirmed by fax.

After patients have been randomised, the investigator should send the patient's general practitioner a letter to inform them that their patient is participating in the study.

## 7.2 Site Responsibilities

The Principal Investigator at each participating centre has overall responsibility for the study and all patients entered into the study, but may delegate responsibility down to other members of the study team as appropriate. The Principal investigator must ensure that all staff involved are adequately trained and their duties have been logged on the Site Responsibilities Log.

## 7.3 Forms and Data Collection

Investigator Folder/s will be supplied containing current CRFs, Patient Consent Forms, Protocol and NCI CTCAE version 3.0. Extra copies of the protocol and CRFs will be downloadable from the BTOG web site at www.btog.org or via email from the **BTOG2 Study Office**. The CRFs must be completed and signed by the investigator or one of their authorised staff members as soon as the requested information is available and the CRF pages returned promptly to the **BTOG2 Study Office**. In all cases it remains the responsibility of the investigator to check that original CRFs are sent to the **BTOG2 Study Office** and to verify that they are completed and filled out correctly.

## 7.4 Protocol Compliance and Monitoring

**BTOG2** is being conducted under the auspices of the Cancer Research UK according to the current guidelines for Good Clinical Practice. All clinical investigators taking part in the trial will be asked to sign the necessary agreements and supply a current CV to the CRCTU. The Principal Investigator will submit Part C of the MREC application to the local Research Ethics Committee (LREC) and will

be responsible for R&D/Trust approval. LREC approval and R&D approval must be received by the BTOG2 Study Office before centre commences recruitment.
The study staff will be in regular contact with centre personnel (by phone/fax/email/letter) to check on progress and any queries that they may have. The **BTOG2 Study Office** will check incoming forms for compliance with the protocol, consistent data, missing data and timing. Investigators will allow the study staff access to source documents as requested. Centres may be withdrawn from further recruitment in the event of serious and persistent non-compliance.

### 7.5 Archiving

All essential source and study documentation must be securely retained by participating centres for at least 15 years in accordance with current regulatory requirements.

## 8) ETHICAL AND REGULATORY STANDARDS

### 8.1 Ethical Conduct of the Study

This study will be carried out in accordance with the World Medical Association (WMA) Declaration of Helsinki (1964) and the Tokyo (1975), Venice (1983), Hong Kong (1989), South Africa (1996) and Scotland (2000) amendments. Copies of the declaration may be obtained by contacting the CRCTU, or from the WMA website: http://www.wma.net/e/policy/17-c_e.html

The protocol will be approved by a Multi-Centre Research Ethics Committee (MREC). Before entering patients into the study, the responsible investigator must ensure that the protocol has the approval of the relevant LREC. The **BTOG2 Study Office** will send an annual trial update report to the MREC, which will be forwarded to each, participating centre, together with details of their individual recruitment.

### 8.2 Regulatory Status

This study will be carried out under a Clinical Trial Authorisation (CTA), and it will be the responsibility on the chief investigator to report SUSARs and SSARs to the regulatory authorities in accordance with EU Directive 2001/20/EC and UK legislation.

### 8.3 Patient Informed Consent

The investigator is required to explain the nature and purpose of the study to the patient prior to study entry. A patient information sheet will be given to the patient and written informed consent obtained before study entry. It is the responsibility of the investigator to obtain written informed consent in compliance with national requirements from each subject prior to entering the trial or, where relevant, prior to evaluating the subject's suitability for the study. The patient information sheet will be available in electronic format from the **BTOG2 Study Office** to enable individual hospitals to put onto their headed paper.

### 8.4 Patient Confidentiality

The personal data recorded on all documents will be regarded as strictly confidential. To preserve the patient’s anonymity, only their initials, date of birth, and hospital number will be recorded on case

report forms after the patient has been randomised into the study. At randomisation with the patient's consent, the patients name and NHS number will be collected and patient's details used to allow flagging with the Office of National Statistics. The Investigator must ensure the patient's anonymity is maintained. The investigator must maintain documents not for submission to the trials unit in strict confidence.

The CRCTU will preserve the confidentiality of patients taking part in this study and will not reproduce or disclose any information by which patients could be identified. Patients should be reassured that their confidentiality will be respected at all times. In the case of special problems and/or governmental queries, it will be necessary to have access to the complete study records, provided that patient confidentiality is protected.

### 8.5 Patient Withdrawal

Patients have the right to withdraw from the study at any time for any reason. The investigator also has the right to withdraw patients from the study. Full details of the reasons for withdrawal should be recorded on the relevant CRF if clinician-initiated; otherwise a simple statement reflecting patient preference will suffice. Withdrawn patients should be followed-up in accordance with the protocol provided patient has no objection.

### 8.6 Protocol Amendment

Any variation in procedure from that specified in the **BTOG2** protocol may lead to the results of the trial being questioned and in some cases rejected. Any proposed protocol change must therefore be submitted in writing to the **BTOG2 Study Office** to be pre-approved by the **BTOG2 Steering Committee**. All agreed protocol amendments will be documented by the CRCTU and will be submitted to the MREC for approval prior to submission to all centres. Changes not pre-approved by the **BTOG2 Steering Committee** will be considered as protocol deviations. This does not affect the individual clinician's responsibility to take immediate action if thought necessary to protect the health and interests of individual patient.

### 8.7 Sponsorship and Indemnity

**BTOG2** is an investigator led and designed trial, co-ordinated by the CRCTU in Birmingham. The University of Birmingham will act as a co-sponsor, whilst Royal Wolverhampton Hospitals NHS Trust is the other co-sponsor, providing the Chief Investigator. The CRCTU does not hold insurance against claims for compensation for injury caused by participation in a clinical trial and they cannot offer any indemnity. As this is a clinician-initiated study, the Association of the British Pharmaceutical Industry (ABPI) guidelines for patient compensation by the pharmaceutical industry will not apply. However, in terms of liability, NHS Trust and Non-Trust Hospitals have a duty of care to patients treated, whether or not the patient is taking part in a clinical trial. Therefore compensation is available in the event of clinical negligence being proven.

## 9) PUBLICATION POLICY

Those centres with the highest level of recruitment will be co-authors on the final publication, along with the named protocol investigators. Other contributors will also be acknowledged.

## 10) REFERENCES


1 Mattson, M.E., Pollack, E.S. and Cullen, J.W. What are the odds smoking will kill you? Am J Public Health . 77: 425-432, 1987.

2 Eisenhauer, E. A., O'Dwyer, P. J., Christian, M., and Humphrey, J. S. Phase I clinical trial design in cancer drug development, Journal of Clinical Oncology. 18: 684-92, 2000.

3 Go, R. S. and Adjei, A. A. Review of the Comparative Pharmacology and Clinical Activity of Cisplatin and Carboplatin, J Clin Oncol. 17: 409-3399, 1999.

4 Campos, D., Pereira, J. R., Reinhardt, R. R., Carracedo, C., Poli, S., Vogel, C., Martinez-Cedillo, J., Erazo, A., Wittreich, J., Eriksson, L.-O., Carides, A. D., and Gertz, B. J. Prevention of Cisplatin-Induced Emesis by the Oral Neurokinin-1 Antagonist, MK-869, in Combination With Granisetron and Dexamethasone or With Dexamethasone Alone, J Clin Oncol. 19: 1759-3739, 2001.

5 Legha, S. and Dimery, I. High-dose cisplatin administration without hypertonic saline: observation of disabling neurotoxicity, J Clin Oncol. 3: 1373-878, 1985.

6 Horwich, A., Sleijfer, D., Fossa, S., Kaye, S., Oliver, R., Cullen, M., Mead, G., de Wit, R., de Mulder, P., Dearnaley, D., Cook, P., Sylvester, R., and Stenning, S. Randomized trial of bleomycin, etoposide, and cisplatin compared with bleomycin, etoposide, and carboplatin in good-prognosis metastatic nonseminomatous germ cell cancer: a Multi-institutional Medical Research Council/European Organization for Research and Treatment of Cancer Trial, J Clin Oncol. 15: 1844-1852, 1997.

7 Kelsen, D. and Atiq, O. T. Therapy of upper gastrointestinal cancers, Curr Prob Cancer. 15: 237-294, 1991

8 Petrioli, R., Frediani, B., Manganelli, A., and al, e. Comparison between a cisplatin containing regimen and a carboplatin containing regimen for recurrent or metastatic bladder cancer patients: A randomised phase II study, Cancer. 77: 344-351, 1996.

9 Non-small cell lung cancer Group. Chemotherapy in non-small cell lung cancer: a meta analysis using updated data on individual patients from 52 randomised clinical trials, Brit Med Journal. 311: 899-909, 1995.

10 Rossoff, A. H., Bearden, J. D., and Coltman, C. A. Phase II evaluation of cis-diamminedichloroplatinum(II) in lung cancer, Cancer Treatment Reports. 60: 1679-1680, 1976.

11 Gandara, D., Crowley, J., Livingston, R., Perez, E., Taylor, C., Weiss, G., Neefe, J., Hutchins, L., Roach, R., and Grunberg, S. Evaluation of cisplatin intensity in metastatic non-small cell lung cancer: a phase III study of the Southwest Oncology Group, J Clin Oncol. 11: 873-878, 1993.

12 Sculier, J., Klastersky, J., Giner, V., Bureau, G., Thiriaux, J., Dabouis, G., Efremidis, A., Ries, F., Berchier, M., and Sergysels, R. Phase II randomized trial comparing high-dose cisplatin with moderate- dose cisplatin and carboplatin in patients with advanced non-small-cell lung cancer. European Lung Cancer Working Party, J Clin Oncol. 12: 353-, 1994.

13 Gralla, R. J., Casper, E. S., Kelsen, D. P., Braun, D. W., Dukeman, M. E., Martini, M., Young, C. W., and Golbey, R. B. Cisplatin and vindesine combination chemotherapy for advanced carcinoma of the lung: A randomised trial investigating two dosage schedules, Annals of Internal Medicine. 95: 414-420, 1981.

14 J Klastersky, JP Sculier, P Ravez, P Libert, J Michel, G Vandermoten, P Rocmans, Y Bonduelle, M Mairesse, and T Michiels A randomized study comparing a high and a standard dose of cisplatin in combination with etoposide in the treatment of advanced non-small cell lung carcinoma JCO 1780-1786, 1986.

15 Shinkai T, Saijo N, Eguchi K, Sasaki Y, Tominaga K, Sakurai M, Suga J, Miyaoka H, Sano T, Keicho N, Takahashi H, Ishihara J, Tamura T, Hoshi A, Jett JR : Cisplatin and vindesine combination chemotherapy for non-small cell lung cancer : a randomized trial comparing two dosages of cisplatin. Jpn J Cancer Res; 77: 782-89, 1986.

16 Raftopoulos, H., Gralla, R.J., D'Auria, G., Neli, F., Bria, E.,
Assessing the role of cisplatin dose and dose intensity (DI) in advanced non-small cell lung cancer(NSCLC): A comprehensive review of randomised trials 1992-2002 Proceedings Am Soc Oncol 22, abstract 2596, 2003

17 Einhorn, L., Loehrer, P., Williams, S., Meyers, S., Gabrys, T., Nattan, S., Woodburn, R., Drasga, R., Songer, J., and Fisher, W. Random prospective study of vindesine versus vindesine plus high-dose cisplatin versus vindesine plus cisplatin plus mitomycin C in advanced non-small-cell lung cancer, J Clin Oncol. 4: 1037-3399, 1986.

18 Cullen, M. H., Billingham, L. J., Woodroffe, C. M., Cheityawardana, A. D., Gower, N. H., Joshie, R., Ferry, D. R., Rudd, R. M., Spiro, S. G., Cook, J. E., Trask, C., Bessell, E., Connolly, C. K., Tobias J, Souhami RL: Mitomycin, ifosfamide and cisplatin in unresectable non-small cell lung cancer: effects on survival and quality of life, J Clin Oncol. 17: 3188-3194, 1999.

19 Crino, L., Scagliotti, G. V., Ricci, S., De Marinis, F., Rinaldi, M., Gridelli, C., Ceribelli, A., Bianco, R., Marangolo, M., Di Costanzo, F., Sassi, M., Barni, S., Ravaioli, A., Adamo, V., Portalone, L., Cruciani, G., Masotti, A., Ferrara, G., Gozzelino, F., and Tonato, M. Gemcitabine and Cisplatin Versus Mitomycin, Ifosfamide, and Cisplatin in Advanced Non-Small Cell Lung Cancer: A Randomized Phase III Study of the Italian Lung Cancer Project, J Clin Oncol. 17: 3522-3092, 1999.

20 Depierre, A., Lemarie, E., Dabois, G., Garnier, G., Jacoulet, P., Dalphin, J. C.: A phase II study of navelbine (vinorelbine) in the treatment of non-small cell lung cancer, Am J Clin Oncol. 14: 15-20, 1991.

21 Millward, M., Zalcberg, J., Bishop, J., Webster, L., Zimet, A., Rischin, D., Toner, G., Laird, J., Cosolo, W., Urch, M., Bruno, R., Loret, C., James, R., and Blanc, C. Phase I trial of docetaxel and cisplatin in previously untreated patients with advanced non-small cell lung cancer, J Clin Oncol. 15: 750-1767, 1997.

22 Murphy, W. K., Fossella, F. V., Winn, R. J., Shin, D. M., Hynes, H. E., Gross, H. M., Davilla, E., Leimert, J., Dhingra, H., Raber, M. N., Krakoff, I. H., Hong, W. K.: Phase II study of taxol of patients with untreated non-small cell lung cancerancer Inst, J Natl C. 85: 384-388, 1993.

23 Anderson, H., Lund, F., Bach, F., Thatcher, N., Walling, J., and Hansen, H. H. Single-agent activity of weekly gemcitabine in advanced non-small cell lung cancer: a phase II study, J Clin Oncol. 12: 1821-1826, 1994.

24 Sandler, A. B., Nemunaitis, J., Denham, C., von Pawel, J., Cormier, Y., Gatzemeier, U., Mattson, K., Manegold, C., Palmer, M. C., Gregor, A., Nguyen, B., Niyikiza, C., and Einhorn, L. H. Phase III Trial of Gemcitabine Plus Cisplatin Versus Cisplatin Alone in Patients With Locally Advanced or Metastatic Non-Small-Cell Lung Cancer, J Clin Oncol. 18: 122-3399, 2000.

25 Masuda, N., Fukuoka, M., Kudoh, S., Kusunoki, Y., Matsui, K., Nakagawa, K., Hirashima, T., Tamanoi, M., Nitta, T., and Yana, T. Phase I study of irinotecan and cisplatin with granulocyte colony- stimulating factor support for advanced non-small cell lung cancer, J Clin Oncol. 12: 90-1662, 1994

26 Klastersky, J., Sculier, J. P., Lacroix, H., Dabouis, G., Bureau, G., Libert, P., Richez, M., Ravez, P., Vandermoten, G., and Thiriaux, J. A randomised study comparing cisplatin or carboplatin with etoposide in patients with advanced non-small cell lung cancer:EORTEC protocol 07861, J Clin Oncol. 8: 1556-1562, 1990.

27 Rudd, R.M., Gower, N.H., James, L.E., Gregory, W., Eisen, T., Lee, S.M., Harper, P.G., Spiro, S.G.: Phase III randomised comparison of gemcitabine plus carboplatin versus mitomycin ifosfamide and cisplatin in advanced non small cell lung cancer. Proc ASCO vol 21, 1164, 2002.

28 Danson, S., Middleton, M.R., O'Byrne, K.J., Clemens, M., Ranson, M., Hassan, J., Anderson, H., Burt, P., Fairve-Finn, C, Stout, R., Dowd, I., Ashcroft, L., Beresford, C., Thatcher, N. Phase III trial of gemcitabine carboplatin versus cisplatin combination chemotherapy in advanced NSCLC. (Brit J Cancer,in press) (2003)

29 Rosell, R., Gatzmeier, U., Betticher, D.C.,Keppler, U., Macha, H.N., Pirker, R., Berthet, P., Breau, J.L., Laines, P.,Nicholson, M., Ardizzoni, A., Chemaissani, A., Bogaerts, J., and Gallant, G. Phase III randomised trial comparing paclitaxel/carboplatin with paclitaxel/cisplatin in patients with advanced non-small cell lung cancer: a cooperative multinational trial Annals of Oncology, 13, 1539-1549, 2002.

30 Belani CP; TAX 326 Study Group. Docetaxel in combination with platinums (cisplatin or carboplatin) in advanced and metastatic non-small cell lung cancer. Semin Oncol, 29 (3 Suppl 12): 4-9, 2002

31 Mazzanti, P., Lippe, P., Battelli, N., Mattioli, R., Buzzi, F., Trivisonne, R., Tuveri, G., Barni, S., De Signoribus, G., Rossi, G., Di Lullo, L., and Battelli, T. Gemcitabine-cisplatin (GP) vs gemcitabine-carboplatin (GC) in advanced non-small cell lung cancer (ANSCLC): A multicenter phase II randomized trial of a 21-day schedule. Preliminary results. Proceedings of the American Society of Clinical Oncology - Meeting 19(36), 540a. 2000.

32 Novakova, L., Novakova, L., Petruzelka, L., Zemanova, M., Kolek, V., Grygarkova, I., Sixtova, D., Roubec, J., Horenkova, E., Kuta, M., Havel, L., and Prusa, P. Gemcitabine plus cisplatin (GCis) versus gemcitabine plus carboplatin (GCarb) in patients with stage IIIB and IV non-small cell lung cancer (NSCLC): Final results of Czech lung cancer cooperative group phase III randomized trial. Proceedings of the American Society of Clinical Oncology 1125(PART 1), 1225a. 2002.

33 Shepherd, F. A. Chemotherapy for Advanced Non-Small-Cell Lung Cancer: Modest Progress, Many Choices, J Clin Oncol. 18S: 35-38, 2000.

34 Hotta, K., Matsuo, K., Ueoka, Hiroshi., Kiura, K., Tabata, M. and Tanimoto, M. Meta-Analysis of randomised clinical trials comparing cisplatin to carboplatin in patients with advanced non-small cell lung cancer. J Clin Oncol. 22(19) 3852-3859, 2004.

35 Wright JG, Boddy AV, Highley M, Fenwick J, McGill A, Calvert AH. Estimation of glomerular filtration rate in cancer patients.Br J Cancer. 2001 Feb;84(4):452-459

# APPENDIX 1) CISPLATIN

SYNONYM(S): Cis-platinum, cis-diamminedichloroplatinum, CDDP, DDP, CIS, Platinum
COMMON TRADE NAME(S): Platinol®, Platinol AQ®
CLASSIFICATION: Alkylating agent

---

## INTRODUCTION

Cisplatin or more accurately cis-diamminedichloroplatinum(II) is one of a number of platinum coordination complexes with anti-tumour activity The compound was first synthesized by M. Peyrone in 1844, but it wasn't until the 1960's; the potential of this compound as an anti-tumour agent was recognised through an observation made by Barnett Rosenberg and co-workers. Their study had been designed to explore the possible effects of an electric field on the growth of Eschericia coli. They observed the bacteria growing 300 times their normal length and ceasing to divide. It was not until a year later that the cause of the inhibition of bacterial division was pinpointed to an electrolysis product of the platinum electrode. This was found to be cisplatin and led to cisplatin being tested against tumours in mice, and the discovery that it is highly effective in eliminating tumours. Human trials followed and the trials culminated in 1978 in the United States with approval for the use of cisplatin in the treatment of testicular and ovarian cancers, and later to bladder cancer. Cisplatin is now widely prescribed for a variety of tumours (germ-cell, advanced bladder carcinoma, adrenal cortex carcinoma, breast cancer, head and neck carcinoma, lung carcinoma

## INDICATIONS

Cisplatin (Platinol®, Platinol®-AQ) is licensed for use in: metastatic testicular tumours, metastatic ovarian tumours, lung carcinomas and advanced bladder cancer.

## MODE OF ACTION

Cisplatin is believed to kill cancer cells by binding to DNA and interfering with its repair mechanism, eventually leading to cell death. Cisplatin enters cells by diffusion, where it is converted to its active form. This is due to the lower intracellular chloride concentration, which promotes ligand exchange of chloride for water, and thus formation of the aquated complex. The aquation of cisplatin is thought to produce the active species, however this has not yet been determined with great certainty. There have been reports that the monoaquated species is the active form, which is dependent on pH - existing as the hydroxy form in basic medium, and proposals of a platinum dimer that is bridged by two hydroxyl groups $[(NH_3)_2Pt\ (\mu\text{-}OH\ )_2Pt(NH_3)_2]^{2+}$ which forms prior to DNA interaction. However, the most common structure is considered to be the diaquated form, cis-diaquadiammineplatinum(II). The principle function of cisplatin is to bind to DNA. The consequence of this is the activation of repair processes, which eventually cause cell death. This explains why cisplatin is sometimes classed as an alkylating agent. Currently, the precise mechanisms that induce cellular apoptosis is not yet fully understood, however, some progress and insights have been made.

## CONTRAINDICATIONS

Contraindications include pregnancy and breast-feeding, pre-existing renal impairment, myelosuppression, hearing impairment and prior allergic reactions to platinum–containing compounds.

## PHARMACOKINETICS

| | | |
|---|---|---|
| Oral Absorption | no | |
| Distribution | Widely distributed with highest levels in kidney, liver and intestines; found in breast milk, distributes into third spaces such as ascites and pleural fluid, may cross the placenta | |
| | Cross blood brain barrier? | trace |
| | Vd | 0.17-1.47 L/kg |
| | PPB | Cisplatin: not significantly<br>inactive metabolites: 90% |
| Metabolism | nonenzymatically transformed to multiple metabolites | |
| | active metabolite(s) | yes (free, filterable platinum) |
| | inactive metabolite(s) | yes |
| Excretion | primarily in urine | |
| | urine | fraction depends on length of infusion |
| | t½ α | 6-13 minutes |
| | t½ β | 25-49 minutes |
| | t½ γ | 2-96 hours |
| | Cl | 6.3 mL/min/kg |

(Table above from Cancer Drug Manual© 1994)

## PRECAUTIONS

- Needles or intravenous administration sets containing aluminium parts that may come in contact with cisplatin should not be used for preparation or administration of the drug. Aluminium can react with cisplatin causing precipitate formation and loss of potency.
- Peripheral blood counts should be monitored weekly, liver function should be monitored periodically, and neurological examination should also be performed regularly.
- Plasma levels of anticonvulsant agents may become sub-therapeutic during cisplatin therapy.
- In a randomised trial in advanced ovarian cancer, response duration was adversely affected when pyridoxine was used in combination with altretamine (hexamethylmelamine) and cisplatin.
- Cisplatin has been shown to have mutagenic and carcinogenic properties in experimental models. Its safe use in pregnancy has not been established. Present in breast milk, therefore, breast-feeding is not recommended.
- Paediatric use, safety and effectiveness in paediatric patients have not been established

## ADVERSE EVENTS

The incidences of adverse events are based on information regarding cisplatin from the Bristol Myers Squibb clinical trials database.

### Summary of Adverse Events

| SUMMARY OF ADVERSE EVENTS | |
|---|---|
| ONSET | SIDE EFFECT<br>$^{LT}$ may be life-threatening; side effects in **bold** type are common |
| IMMEDIATE (hours to days) | $^{LT}$ anaphylaxis (1-20%) |
| | **nausea and vomiting**<br>(most patients, moderate to severe, onset 1-4 hours, duration 1-7 days) |
| EARLY (days to weeks) | $^{LT}$ low WBC, RBC, platelets (25-30%, myelosuppression, nadir 18-23 days, recovery 39 days) |
| | **kidney problems** (28-36%, toxic nephropathy, hypomagnesemia, electrolyte disturbances) |
| | nausea and vomiting |
| | heart problems (electrocardiographic changes, rare) |
| | liver problems (elevated liver function tests, rare) |
| | blood problems (hemolytic anemia) |
| | CNS problems (acute encephalopathy, rare) |
| DELAYED/LATE (weeks to years) | **nerve problems** (peripheral neuropathy) |
| | CNS problems (acute encephalopathy, rare) |
| | eye problems (retinopathy, optic neuropathy) |
| | hearing problems (24%, ototoxicity) |
| | infertility |
| | Raynaud's syndrome (rare) |

(Table above from Cancer Drug Manual© 1994)

**Nephrotoxicity:** Dose-related and cumulative renal insufficiency is the major dose-limiting toxicity of cisplatin. Renal toxicity has been noted in 28% to 36% of patients treated with a single dose of 50mg/m$^2$. It is first noted during the second week after a dose and is manifest by elevations in blood urea nitrogen and Creatinine, serum uric acid and/or a decrease in Creatinine clearance.

**Ototoxicity**: Ototoxicity has been observed in up to 31% of patients treated with a single dose of cisplatin 50mg/m$^2$ and is manifested by tinnitus and/or hearing loss in the high frequency range (4000 to 8000 Hz). Decreased ability to hear normal conversational tones may occur occasionally. Deafness after the initial dose has been reported rarely. Hearing loss can be unilateral or bilateral and tends to become more frequent and severe with repeated doses. It is unclear whether cisplatin induced ototoxicity is reversible.

**Haematological**: Myelosuppression is seen in 25% to 30% of patients, with nadirs in circulating platelets occurring between days 18-23 and most patients recovering by day 39. Leukopenia and thrombocytopenia are more pronounced at higher doses; anaemia occurs at approximately the same frequency and with the same timing. Fever and infection have also been reported in patients with neutropenia.
In addition to anaemia secondary to myelosuppression, a Coombs' positive haemolytic anaemia has been reported.

The development of acute leukaemia coincident with cisplatin has rarely been reported in humans. In these reports cisplatin was generally given with other leukemogenic agents

**Gastrointestinal:** Marked nausea and vomiting occur in almost all patients treated with cisplatin and are occasionally so severe that the drug must be discontinued. Nausea and vomiting usually begin within 1 to 4 hours after treatment and lasts up to 24 hours. Various degrees of vomiting, nausea and/or anorexia may persist for up to 1 week after treatment. Delayed nausea and vomiting (begins or persists 24 hours or more after chemotherapy has occurred in patients attaining complete emetic control on the day of cisplatin therapy. Diarrhoea has been reported.

**OTHER TOXICITIES**

**Serum Electrolyte Disturbances:** Hypomagnesaemia, hypocalcaemia, hyponatraemia, hypokalaemia and hypophosphataemia have been reported to occur and are probably related to renal tubular damage. Tetany has occasionally been reported in those patients with hypocalcaemia and Hypomagnesaemia. Generally normal serum electrolyte levels are restored by administering supplemental electrolytes and discontinuing cisplatin. In appropriate anti-diuretic hormone syndrome has also been reported.

**Hypersensitivity:** Occasionally reported in patients exposed to cisplatin. Contraindicated in patients with a history of allergic reactions to cisplatin or other platinum-containing compounds. Anaphylactic-like reactions occurring within minutes of administration seen with prior exposure to cisplatin, and have been relieved with use of epinephrine, corticosteroids, and antihistamines.

**Hyperuricaemia:** Has been reported to occur at approximately same frequency as the increases in blood urea nitrogen and serum creatinine. Hyperuricaemia is more pronounced after doses > 50mg/m$^2$, with peak levels occurring generally between 3-5 days after dose. Allopurinol therapy effectively reduces uric acid levels.

**Neurotoxicity:** Neurotoxicities usually characterised by peripheral neuropathies has been reported. The neuropathies usually occur after prolonged therapy (4 to 7 months), however neurological symptoms have been reported to occur after a single dose. Although symptoms and signs of cisplatin neuropathy usually develop during treatment, symptoms may begin 3 to 8 weeks after the last dose of cisplatin, although this is rare. The neuropathy may progress further even after stopping treatment. Preliminary evidence suggests peripheral neuropathy may be irreversible in some patients.

Lhermitte's sign, dorsal column myelopathy and autonomic neuropathy have also been reported. Loss of taste and seizures has also been reported.

Muscle cramps defined as localised, painful, involuntary skeletal muscle contractions of sudden onset and short duration have been reported and were usually associated in patients receiving relatively high cumulative dose of cisplatin and with a relatively advanced symptomatic stage of peripheral neuropathy.

**Ocular toxicity**: Optic neuritis, papilloedema and cerebral blindness have been reported infrequently in patients receiving standard recommended doses of cisplatin. Improvement and/or total recovery usually occurs after discontinuing cisplatin.

Blurred vision and altered colour perception manifests as a loss of colour discrimination, particularly in the blue-yellow axis. The only finding on funduscopic exam is irregular retinal pigmentation of the macular area.

**Hepatotoxicity** Transient elevations of liver enzymes, especially SGOT, as well as bilirubin have been reported to be associated with cisplatin administration at the recommended doses.

**Other Events** Other toxicities reported to occur infrequently are cardiac abnormalities, hiccups, elevated serum amylase and rash. Alopecia, malaise, and asthenia have been reported.

Local soft tissue toxicity has rarely been reported following extravasation of cisplatin. Severity of the local tissue toxicity appears to be related to the concentration of the cisplatin solution. Infusion of solutions with a concentration >0.5mg/mL may result in tissue cellulites, fibrosis and necrosis.

There are serious side effects associated with cisplatin, notably renal toxicity, emesis, neurotoxicity, bone marrow suppression and hearing loss. Damage to the kidneys can be minimized through the administration of continuous IV hydration along with diuretic drugs before and following the infusion of cisplatin. Similarly, several effective anti-emetic drugs protect the patient from the worst of nausea and vomiting. Testing of patient renal function, blood and hearing is recommended before each cycle of therapy

## INTERACTIONS

| AGENT | EFFECT | MECHANISM | MANAGEMENT |
|---|---|---|---|
| renally excreted drugs (especially ifosfamide, high dose methotrexate) | Decreased renal clearance and increased t½; toxicities of these drugs may be enhanced | reduced renal function caused by cisplatin | Ascertain renal function prior to giving potentially toxic really-excreted drugs (such as other chemotherapy) and modify doses as necessary |
| aminoglycosides amphotericin | Increased nephrotoxicity | Additive | Use with extreme caution during or shortly after cisplatin therapy |
| etoposide | Synergistic effect against certain tumours when combined with cisplatin (testicular cancer, lung cancer) | Possibly by decreased clearance of etoposide | Some protocols are designed to take advantage of this effect |
| etoposide | Improved survival in lung cancer patients when cisplatin given before etoposide | Unknown | Give cisplatin before etoposide |
| Furosemide ethacrynic acid | Increased ototoxicity | Additive | avoid concomitant use; use furosemide if a diuretic is essential (may be less ototoxicthan ethacrynic acid) |
| phenytoin | Decreased phenytoin serum levels | Decreased absorption and/or increased metabolism of phenytoin | Monitor for decreased anticonvulsant effects and phenytoin serum levels; increase phenytoin dose if necessary |

Table above from Cancer Drug Manual© 1994

# APPENDIX 2) CARBOPLATIN

SYNONYM(S): CBDCA, JM8, NSC 241240
COMMON TRADE NAME(S): Paraplatin®, Paraplatin AQ®
CLASSIFICATION: Alkylating agent, cytotoxic

## INTRODUCTION

The search for a less toxic agent than cisplatin was pursued at the Institute for Cancer Research in the United Kingdom and led to the development of carboplatin. Carboplatin or more accurately diammine 1,1-cyclobutane-dicarboxylato platinum (II), entered clinical trials in 1981 and showed a very similar activity profile to that of cisplatin, with good response in ovarian, small cell lung, head and neck, and testicular cancers. It is now currently the second most widely used platinum anticancer drug in the world.

## INDICATIONS

Carboplatin (*Paraplatin®, Paraplatin®-AQ)* is licensed for use in: advanced ovarian carcinoma and small cell lung cancer.

## MODE OF ACTION

Carboplatin like cisplatin produces predominantly inter-strand DNS cross-links rather than DNA-protein cross-links. The effect is apparently cell cycle non-specific. The aquation of carboplatin, which is thought to produce the active species, occurs at a slower rate than in the case of cisplatin. Despite this difference it appears to induce equal numbers of drug-DNA cross-links as cisplatin causing equivalent lesions and biological effects

## CONTRAINDICATIONS

Contraindications include pregnancy and breast-feeding, patients with severe bone marrow depression or significant bleeding, history of severe allergic reactions to platinum-containing compounds, or mannitol.

## PHARMACOKINETICS

| | | |
|---|---|---|
| Interpatient variability | 2- to 3-fold variability in AUC with BSA-based dosing. Variability can be reduced with Calvert AUC-based dosing formula) | |
| Oral Absorption | Poorly absorbed; oral route not used clinically) | |
| Intraperitoneal Absorption | Peak plasma level within 2-4 h after intraperitoneal instillation with 65% of dose absorbed over 4 h of dwelling | |
| Distribution | Widely distributed, mostly in kidney, liver, skin, tumour tissue; also in erythrocytes | |
| | Cross blood brain barrier? | yes |
| | Volume of distribution | ultrafilterable platinum* : 17 ± 2 L/1.73 $m^2$ |
| | Plasma protein binding | Carboplatin: minimal Platinum: 87% |
| Metabolism | Undergoes intracellular hydrolysis to form reactive platinum complexes | |

| | | |
|---|---|---|
| | Active metabolite(s) | Platinum complexes |
| | Inactive metabolite(s) | no information found |
| Excretion | Renal excretion via glomerular filtration; extensively removed by hemodialysis. | |
| | Urine | 71% within 24 h |
| | Terminal half life | 5.8 ± 1.6 days (total platinum*) Platinum elimination from erythrocytes: 12 days |
| | Clearance | 1.38 ± 0.36 L/h/1.73 m$^2$ (total platinum*)) |
| Gender | No information found | |
| Elderly | Clearance may be reduced due to age-related renal function impairment | |
| Children | Similar to adults) | |
| Ethnicity | No information found | |

From Cancer Drug Manual© 2001

## PRECAUTIONS

- Needles or intravenous administration sets containing aluminium parts that may come in contact with carboplatin should not be used for preparation or administration of the drug. Aluminium can react with carboplatin causing precipitate formation and loss of potency;
- Prior exposure to cisplatin: increases the risk and severity of toxicities (e.g. myelosuppression, nausea, vomiting, peripheral neuropathy, ototoxicity);
- Carcinogenicity: has not been fully studied, but drugs with similar mechanisms of action and mutagenicity have been reported to be carcinogenic;
- Mutagenicity: mutagenic in both in vitro and in vivo studies;
- Fertility: may cause gonadal suppression (amenorrhea, azoospermia), which is generally related to dose and length of therapy and may be irreversible;
- There is positive evidence of human fetal risk, but the benefits from use in pregnant women may be acceptable despite the risk (e.g., if the drug is needed in a life-threatening situation or for a serious disease for which safer drugs cannot be used or are ineffective);
- Breastfeeding is not recommended due to the potential secretion into breast milk;
- Paediatric use, safety and effectiveness in paediatric patients have not been established.

## ADVERSE EVENTS

The incidences of adverse events are based on information regarding carboplatin from the Bristol Myers Squibb clinical trials database.

**Haematological:** Myelosuppression is the dose-limiting toxicity of carboplatin. Thrombocytopenia with platelet counts below 50,000/mm$^3$ occurs in 25% of the patients (35% of pre-treated ovarian cancer patients).; neutropenia with granulocyte counts below 1000/mm$^3$ occurs in 16% of the patients (21% of pre-treated ovarian cancer patients): leukopenia with WBC counts below 2000/mm$^3$ occurs in 15% of the patients (26% of pre-treated ovarian cancer patients). The nadir usually occurs about day 21 in patients receiving single-agent therapy. By day 28, 90% of patients have platelet counts above 100,000mm$^3$, 74% have neutrophil counts above 2,000/mm$^3$, and 67% have leukocyte counts above 4,000/mm$^3$.

Myelosuppression is usually more severe in patients with impaired kidney function. Patients with poor performance status have also experienced a higher incidence of severe leukopenia and thrombocytopenia.

## Summary of Adverse Events

| ORGAN SITE | SIDE EFFECT | ONSET | | | |
|---|---|---|---|---|---|
| Dose-limiting side effects are in ***bold, italics***<br>I = immediate (onset in hours to days); E = early (days to weeks);<br>D = delayed (weeks to months); L = late (months to years) | | | | | |
| allergy/immunology | hypersensitivity (2-30%) | I | E | | |
| auditory/hearing | ototoxicity (tinnitus, visual and taste disturbances) (1%) | | E | | |
| blood/bone marrow<br>febrile neutropenia | ***anemia*** (71%) | | E | | |
| | ***leukopenia*** (severe 14%)<br>nadir 21 days; recovery 30 days[1] | | E | | |
| | ***neutropenia*** (severe 18%)<br>nadir 21-28 days; recovery 35 days | | E | | |
| | ***thrombocytopenia*** (severe 25%)<br>nadir 21 days; recovery 30 days | | E | | |
| constitutional symptoms | asthenia (8%) | | E | | |
| dermatology/skin | *extravasation hazard:* nonvesicant | I | | | |
| | alopecia (3%) | | | D | |
| gastrointestinal | *emetogenic potential:* high moderate | | | | |
| | constipation (6%) | | E | | |
| | diarrhea (6%) | | E | | |
| | nausea (15%) | I | | | |
| | vomiting (64%) | I | | | |
| hepatic | elevated alkaline phosphatase (24%) | | E | | |
| | elevated AST (15%) | | E | | |
| | elevated bilirubin (5%) | | E | | |
| infection | infections (4%) | | E | | |
| metabolic/laboratory | hypocalcemia (22%) | | E | | |
| | hypomagnesemia (29%) | | E | | |
| | hypokalemia (20%) | | E | | |
| | hyponatremia (29%) | | E | | |
| | increased BUN (14%) | | E | | |
| | increased uric acid (5%) | | E | | |
| neurology | CNS symptoms (5%) | | E | | |
| | peripheral neuropathy (4%) | | E | | |
| ocular/visual | visual disturbances (rare)[2] | | E | D | |
| pain | abdominal pain (17%) | I | E | | |
| renal/genitourinary | acute renal failure (rare) | | E | | |
| | decreased creatinine clearance (27%) | | E | | |
| | Increased serum creatinine (6%) | | E | | |
| syndromes | hemolytic-uremic syndrome (rare) | | E | | |

From Cancer Drug Manual© 2001.

The haematological effects although usually reversible have resulted in infectious or hemorrhagic complications in 5% of patients, with drug related death occurring in less than 1% of patients. Fever has also been reported in patients with neutropenia.

Anaemia with haemoglobin less than 11g/dL has been observed in 71% of patients who started therapy with a baseline above that value. The incidence of anaemia increases with increasing exposure to carboplatin.

Myelosuppression maybe more severe when carboplatin is combined with other myelosuppressive drugs or radiotherapy.

**Gastrointestinal:** Vomiting occurs in 65% of the patients and in about one third of these patients it is severe. Nausea alone occurs in 10% to 15% of patients. Both nausea and vomiting usually cease with 24 hours of treatment and are often responsive to anti-emetic measures. Other gastrointestinal effects observed frequently are pain, in 17% of patients, diarrhoea in 6% and constipation in 6%.

**Neurologic toxicity:** Peripheral neuropathies have been observed in 4% of patients receiving cisplatin (6% pretreated ovarian cancer patients) with mild paresthesias occurring most frequently. Clinical ototoxicity and other sensory abnormalities such as visual disturbances and change in taste have been reported in only 1% of the patients. Central nervous system symptoms have been reported in 5% of the patients and appear to be most related to the use of anti-emetics.

Although the overall incidence of peripheral neurologic side effects is low, prolonged treatment may result in cumulative neurotoxicity.

**Nephrotoxicity:** Development of abnormal renal function test results is uncommon, even though administered without high-volume fluid hydration and/or forced diuresis. The incidences of abnormal renal function tests reported are 6% for serum creatinine and 14% for blood urea nitrogen. Most of these reported abnormalities have been mild and about one-half of them were reversible.

**Hepatic Toxicity:** The incidences of abnormal liver function tests in patients with normal baseline values were reported as follows: total bilirubin 5%; SGOT, 15%; alkaline phosphatase, 24%. These abnormalities have generally been mild and reversible in about one-half of the cases.

**Electrolyte Changes:** The incidences of abnormally decreased serum electrolyte values reported were as follows: sodium, 29%; potassium, 20%; calcium, 22%; magnesium, 29%, and were rarely associated with symptoms.

**Allergic Reactions:** Hypersensitivity to carboplatin has been reported in 2% of the patients, i.e. rash, urticaria, erythema, pruritus and rarely bronchospasm and hypotension. Anaphylactic reactions have been reported

**Injection site reactions:** Including redness, swelling and pain have been reported. Necrosis associated with extravasation has also been reported.

**Other events:** Pain and asthenia were the most frequently reported miscellaneous adverse effects; their relationship to tumour or anaemia was likely. Alopecia was reported 3%. cardiovascular, respiratory, genitourinary and mucosal side effects have occurred in 6% or less patients.

## INTERACTIONS

| AGENT | EFFECT | MECHANISM | MANAGEMENT |
|---|---|---|---|
| aminoglycosides (eg, amikacin, gentamycin, tobramycin) | increased risk of carboplatin nephrotoxicity and ototoxicity | Additive | Use with caution during concurrent therapy |
| Phenytoin | Decreased serum phenytoin level | Possibly decreased absorption or increased metabolism of phenytoin | Monitor serum phenytoin level carefully during and after carboplatin therapy; adjust phenytoin dose as needed |
| warfarin | increased anticoagulant effect of warfarin | Unknown; possibly decreased protein binding or decreased metabolism of warfarin | Monitor INR carefully during and after carboplatin therapy; adjust warfarin dose as needed |

From Cancer Drug Manual© 2001.

# APPENDIX 3) GEMCITABINE

SYNONYM(S): Gemcitabine hydrochloride, difluorodeoxycytidine, 2',2'-difluorodeoxycytidine, dFdC, LY 188011

COMMON TRADE NAME(S): Gemzar® (notice of compliance,1 December 1996; patent expires 2 March 2004)

CLASSIFICATION: Antimetabolite, cytotoxic

## INTRODUCTION

Gemcitabine (2'deoxy-2'.2'-difluorocytidine monohydrochloride (dFdC, β isomer)) was initially synthesised as a potential anti-viral drug with excellent activity against both RNA and DNA viruses *in vitro*. However its poor therapeutic index in this setting, and its characterisation as a potent and specific deoxycitidine analogue prompted its subsequent successful evaluation as a cytotoxic drug. After showing broad-spectrum activity in a range of pre-clinical models, gemcitabine entered phase I clinical trials in 1987 using a day 1, day 8 and day 15, q 28 day regimen, and a 30 min infusion.

## INDICATIONS

Gemcitabine (Gemzar®) is licensed for use against non-small cell lung cancer and pancreatic cancer and has been shown to improve quality of life

## MODE OF ACTION

Like other anti-metabolites, gemcitabine exhibits cell phase specificity, primarily killing cells undergoing DNA synthesis (S-phase) and blocking the progression of cells through the G1/S-phase boundary. As a pro-drug, it is phosphorylated by deoxycitidine kinase to dFdC-5'-monophosphate (dFdCMP), then to dFdC5'diphosphate (dFdCDP), and subsequently dFCC-5'triphosphate(dFdCTP). The cytotoxic effect of gemcitabine is attributed to a combination of the two actions of the diphosphate and triphosphate nucleosides, which lead to inhibition of DNA synthesis. The diphosphate first inhibits ribonucleotide reductase, which catalyses the reactions that generate deoxynucleoside triphosphates required for DNA synthesis. This inhibition causes a reduction in the concentration of deoxynucleotide, including dCTP. Then the triphosphate competes with dCTP for incorporation into DNA. The reduction in dCTP concentration enhancing the incorporation of the gemcitabine triphosphate into the DNA. When incorporated into DNA this results in chain termination. DNA polymerase epsilon is unable to remove the gemcitabine nucleotide and repair the growing DNA strands (masked chain termination). In CEM T lymphoblastoid cells, gemcitabine induces internucleosomal DNA fragmentation, one of the characteristics of programmed cell death.

Gemcitabine has also demonstrated dose-dependent synergistic activity with cisplatin. *In vitro* cisplatin had no affect on gemcitabine triphosphate accumulation or DNA double-strand breaks. *In vivo* gemcitabine showed activity in combination with cisplatin against LX-1 and CALU-6 human lung xenografts, but minimal activity was seen with the NCI-H460 or NCI-H520 xenografts. Sequential exposure to gemcitabine 4 hours before cisplatin produced the greatest interaction.

## CONTRAINDICATIONS

Gemcitabine is contraindicated in patients with a known hypersensitivity to the drug.

## PHARMACOKINETICS

| | | |
|---|---|---|
| Interpatient variability | 3- to 4-fold interpatient and intrapatient variability | |
| Oral absorption | No information found | |
| Distribution | Widely distributed into tissues; also present in ascitic fluid. | |
| | Cross blood brain barrier? | no information found |
| | Volume of distribution | IV infusion < 70 min: 50 L/m$^2$;<br>IV infusion 70-285 min: 370 L/m$^2$ |
| | Plasma protein binding | < 10% |
| Metabolism | Metabolized intracellularly by nucleoside kinases to active metabolites dFdCDP and dFdCTP; also metabolized intracellularly and extracellularly by cytidine deaminase to inactive metabolite difluorodeoxyuridine (dFdU). | |
| | Active metabolite(s) | dFdCDP, dFdCTP |
| | Inactive metabolite(s) | dFdU |
| Excretion | Mainly renal excretion | |
| | Urine | 92-98% over one week (89% as dFdU, < 10% as gemcitabine) after a single dose of 1000mg/m$^2$ given over 30 minutes. |
| | Terminal half life | IV infusion < 70 min: 0.7-1.6 h; IV infusion 70-285 min: 4.1-10.6 h |
| | Clearance | IV infusion < 70 min: 41-92 L/h/m$^2$(male), 31-69 L/h/m$^2$ (female) |
| Gender | Decreased volume of distribution and clearance in women | |
| Elderly | Decreased clearance and increased half-life with increasing age | |
| Children | No information found | |
| Ethnicity | No information found | |

(Table above from Cancer Drug Manual© 2001)

## PRECAUTIONS

- Carcinogenicity: No information found;
- Mutagenicity: Not mutagenic in Ames test but mutagenic in mammalian in vitro mutation test. Gemcitabine is clastogenic in mammalian in vitro and in vivo chromosome tests;
- Fertility: Decreased spermatogenesis and fertility in male mice;
- Pregnancy: DA Pregnancy Category D.5. There is positive evidence of human fetal risk, but the benefits from use in pregnant women may be acceptable despite the risk (e.g. if the drug is needed in a life-threatening situation or for a serious disease for which safer drugs cannot be used or are ineffective);
- Breastfeeding is not recommended due to the potential secretion into breast milk;
- Renal and hepatic impairment: the effects of significant renal or hepatic insufficiency on the disposition of gemcitabine have not been assessed. Laboratory evaluation of renal and hepatic function, including transaminases and serum creatinine, should be performed prior to initiation of therapy and periodically thereafter. Gemcitabine should be administered with caution in patients with evidence of significant renal or hepatic impairment;
- Age: Gemcitabine clearance is reduced with age, but there is no evidence that usual dose adjustments are required. Radiotherapy: concurrent or intercalated gemcitabine and radical radiotherapy are to be avoided. However, there is no evidence that radiotherapy following gemcitabine exposure is problematic.

## ADVERSE EVENTS

The incidences of adverse events are based on information regarding gemcitabine (Gemzar®) from the Eli Lily and company clinical trials database

**Summary of Adverse Events**

| ORGAN SITE | SIDE EFFECT | ONSET | | | |
|---|---|---|---|---|---|
| Dose-limiting side effects are in ***bold, italics***<br>I = immediate (onset in hours to days); E = early (days to weeks);<br>D = delayed (weeks to months); L = late (months to years) | | | | | |
| allergy/immunology | allergic reaction (4%, severe 0.2%) | I | | | |
| blood/bone marrow<br>febrile neutropenia | ***Anaemia*** (68%, severe 8%) | | E | | |
| | leukopenia (62%, severe 9%) | | E | | |
| | ***Neutropenia*** (63%, severe 25%)<br>nadir 7-10 days, recovery within 7 days | | E | | |
| | ***thrombocytopenia*** (24%, severe 5%)<br>nadir 7-10 days, recovery within 7 days | | E | | |
| cardiovascular (arrhythmia) | cardiac arrhythmia (2%, severe 0.2%) | | E | | |
| cardiovascular (general) | edema/peripheral edema (28%, severe 3%) | | E | D | |
| coagulation | hemolytic uremic syndrome (0.3%) | | | D | |
| constitutional symptoms | asthenia (42%, severe 2%) | | E | | |
| | fever (37%, severe < 1%) | I | E | | |
| dermatology/skin | *extravasation hazard:* none | | | | |
| | alopecia (14%) | | | D | |
| | skin rash (25%, severe < 1%) | I | E | | |
| gastrointestinal | *emetogenic potential:* low moderate | | | | |
| | constipation (8%, severe < 1%) | | E | | |
| | diarrhea (12%, severe < 1%) | | E | | |
| | nausea and vomiting (64%, severe 18%) | I | | | |
| | stomatitis (8%, severe < 1%) | | E | | |
| hemorrhage | hematuria (31%, severe < 1%) | | E | | |
| hepatic | elevated alkaline phosphatase (55%, severe 9%) | | E | | |
| | elevated AST (67%, severe 9%) | | E | | |
| | elevated ALT (68%, severe 10%) | | E | | |
| | elevated bilirubin (13%, severe 2%) | | E | | |
| infection | infection (9%, severe 1%) | | E | | |
| neurology | decreased level of consciousness (9%, severe < 1%) | | E | | |
| | peripheral neuropathy (3%) | | E | D | |
| pain | pain (16%, severe 1%) | | E | D | |
| pulmonary | dyspnea (8%, severe 1%) | I | E | | |
| renal/genitourinary | elevated BUN (16%, severe 0%) | | E | | |
| | elevated creatinine (7%, severe < 1%) | | E | | |
| | proteinuria (36%, severe < 1%) | | E | | |
| syndromes | flu-like symptoms (19%, severe 1%)[30] | | E | | |

(Table above from Cancer Drug Manual© 2001)

**Haematological:** Myelosuppression is the dose-limiting toxicity with gemcitabine. In pancreatic studies red blood cell transfusions were required by 19% of patients, and the incidence of sepsis

was less than 1%. Petechiae or mild blood loss (haemorrhage), from any cause was reported in 165 of patients; less than 1% of patients required platelet transfusions. Patients should be, monitored for myelosuppression

**Gastrointestinal:** Nausea and vomiting have been commonly reported (69%) but were usually of mild to moderate severity. Severe nausea and vomiting (WHO grade ¾) occurred in <15% of the patients. Diarrhoea was reported by 19% patients, and stomatitis by 115 of patients

**Hepatic:** transient elevations of one or both serum transaminases are seen in approximately 70% of patients

**Cardiotoxicity** Typically 2% of patients treated with single agent gemcitabine have discontinued treatment for myocardial infarction, cerebrovascular accident, arrhythmia, or hypertension, but many of these had prior cardiovascular disease**.**

**Renal:** Mild proteinuria and haematuria are common. Haemolytic uraemic syndrome (HUS) has been reported in 6 of 2429 patients (0.25%) receiving gemcitabine in clinical trials, two of which developed it after cessation of treatment

**Fever :** The overall incidence of fever has been reported as 41%. This is in contrast to the incidence of infection (16%), indicating gemcitabine may cause fever in the absence of clinical infection. Fever was frequently associated with flu-like symptoms and usually mild and clinically manageable.

**Rash:** Rash has been reported in 30% of patients, being typically macular or finely granular maculopapular pruritic eruption of mild to moderate severity involving the trunk and extremities. Pruritus has been reported for 13% of patients.

**Pulmonary:** Dyspnoea was reported in 23% of patients, severe dyspnoea in 3%. Dyspnoea maybe due to underlying disease such as lung cancer (40% of study population) or pulmonary manifestations of other malignancies. Dyspnoea was occasionally accompanied by bronchospasm (< 2% of patients). Rare reports of parenchymal lung toxicity consistent with drug induced pneumonitis have been associated with the use of gemcitabine. Rarely pulmonary oedema of unknown etiology, sometimes severe, has occurred in association with gemcitabine therapy

**Oedema:** Oedema (13%), peripheral oedema (20%) and generalised oedema (<1%) were reported. Less than 1% of patients discontinued due to oedema.

**Flu-like symptoms:** "Flu syndrome" was reported for 19% of patients. Individual symptoms of fever, asthenia, anorexia, headache, cough, chill and myalgia were commonly reported. Fever and asthenia were also reported frequently as isolated symptoms. Insomnia, rhinitis, sweating and malaise are less frequently reported. Less than 1% of patients discontinued treatment due to flu-like symptoms.

**Infection:** Infections were reported for 16% of patients. Sepsis was rarely reported (<1%).

**Alopecia:** Hair-loss is usually minimal, reported by 155 of patients.

**Neurotoxicity:** There was a 10% incidence of mild paresthesias and a <1% rate of sever paresthesias.

**Extravasation:** Injection-site related events were reported for 4% of patients. There were no reports of injection site necrosis.

**Allergic:** Bronchospasm was reported for less than 2% of patients. Anaphylactic reactions have been reported rarely.

**Cardiovascular:** 2% of patients discontinued therapy with gemcitabine due to cardiovascular events such as myocardial infarction, cerebrovascular accident, arrhythmia, and hypertension. Many of these patients had a prior history of cardiovascular disease.

## INTERACTIONS

| AGENT | EFFECT | MECHANISM | MANAGEMENT |
|---|---|---|---|
| warfarin | increased anticoagulant effect of warfarin | possibly decreased metabolism of warfarin and decreased hepatic synthesis of clotting factors | monitor INR carefully during and for 1-2 months after gemcitabine therapy; adjust warfarin dose as needed |

# APPENDIX 4) AMERICAN JOINT COMMITTEE ON CANCER STAGING: LUNG CANCER

## TNM AND STAGING DEFINITIONS

## STAGING

| Stage | T | N | M |
|---|---|---|---|
| Occult | X | 0 | 0 |
| 0 | IS | 0 | 0 |
| Ia | 1 | 0 | 0 |
| Ib | 2 | 0 | 0 |
| IIa | 1 | 1 | 0 |
| IIb | 2 | 1 | 0 |
| | 3 | 0 | 0 |
| IIIa | 3 | 1 | 0 |
| | 1-3 | 2 | 0 |
| IIIb | Any | 3 | 0 |
| | 4 | Any | 0 |
| IV | Any | Any | 1 |

*Ref: Mountain CF - "Revisions in the international system for staging lung cancer", Chest 1997, vol: 111(6) P: 1710-1717.*

## DEFINITIONS

### Primary tumour (T)

TX Tumour proven by the presence of malignant cells in bronchopulmonary secretions but not visualized by roetgenography or bronchoscopy, or any tumour that cannot be assessed in retreatment staging.

T0 No evidence of primary tumour.

TIS Carcinoma in situ.

T1 A tumour that is 3.0 cm or less in greatest dimension, surrounded by lung or visceral pleura, and without evidence of invasion proximal to a lobar bronchus at bronchoscopy*

T2 A tumour more than 3.0 cm in greatest dimension, or a tumour of any size that either invades the visceral pleura or has associated atelectasis or obstructive pneumonitis extending to the hilar region. At bronchoscopy, the proximal extent of demonstrable tumour must be within a lobar bronchus or at least 2.0 cm distal to the carina. Any associated atelectasis or obstructive pneumonitis must involve less than an entire lung.

T3 A tumour of any size with direct extension into the chest wall (including superior sulcus tumours), diaphragm or the mediastinal pleura or percardium without involving the heart, great vessels, trachea, oesophagus or vertbralbody, or a tumour in the main bronchus within 2.0 cm of the carina without involving the carina.

T4 A tumour of any size with invasion of the mediastinum or involving heart, great vessels, trachea, oesophagus, vertebral body or carina or with presence of malignant pleural effusion.**

**Nodal Involvement (N)**

N0 No demonstrable metastasis or regional lymph nodes.

N1 Metastasis to lymph nodes in the peribronchial or the ipsilateral hilar region, or both, including direct extension.

N2 Metastasis to ipsilateral mediastinal lymph nodes and subcarinal lymph nodes.

N3 Metastasis to contralateral mediastinal lymph nodes, contralateral hilar lymph nodes, or ipsilateral or contralatral scalene or supraclavicular lymph nodes.

**Distant Metastasis (M)**

M0 No known metastasis.

M1 Distant metastasis present - specify site(s).

---

* The uncommon superficial tumour of any size whose invasive component is limited to the bronchial wall and that may extend proximal to the main bronchus is classified as T1.

** Most pleural effusions associated with lung cancer are due to tumour. There are however, some few patients in whom cytopathologic examination of pleural fluid (on more than one specimen) is negative for tumour and the fluid is non-bloody and is not an exudate. When these elements and clinical judgement dictate that the effusion is not related to the tumour, the cases should be staged T1, T2 or T3, with effusion being excluded as a staging element.

# APPENDIX 5) WORLD HEALTH ORGANIZATION (WHO) PERFORMANCE STATUS

| Status | Description |
|---|---|
| **0** | Asymptomatic, fully active and able to carry out all pre disease performance without restrictions |
| **1** | Symptomatic, fully ambulatory but restricted in physically strenuous activity and able to carry out performance activity of a light or sedentary nature |
| **2** | Symptomatic, ambulatory and capable of self-care but unable to carry out any work activities. Up and about 50% of waking hours: in bed less than 50% of the day |
| **3** | Symptomatic, capable of only limited self care, confined to bed or chair more that 50% of waking hours, but not bed ridden |
| **4** | Completely disabled. Cannot carry out any self care. Totally bedridden . |

# APPENDIX 6) FORMULAE

## CALVERT FORMULA

**Carboplatin dose = AUC x {(calculated GFR) + 25}.**

*Ref: Calvert AH, Newekk DR, Gumbrell LA, et al. Carboplatin dosage: prospective evaluation of a simple formula based on renal function. J Clin Oncol 1989; 7:1748-5*

The Wright[35] paper is available in section 8.3 of the Investigator Folder.

## WRIGHT FORMULAE

The BTOG2 office will supply "GFR calculators" to centres in both electronic (Excel spreadsheet) and paper formats but the precise version of this spreadsheet depends on which method of serum creatinine measurement is used

### WRIGHT EQUATION: ENZYMATIC

$$\mathrm{GFR} = \frac{(4350 - 34 \times \mathrm{Age} + 522 \times \mathrm{Ln(CK)}) \times \mathrm{BSA} \times (1 - 0.217 \times \mathrm{Sex})}{\mathrm{SCr}}$$

| | | | | | |
|---|---|---|---|---|---|
| **BTOG2 Trial** | | | | | |
| **Calculation of BSA, GFR and Carboplatin Dose** | | | | | |
| **Using Wright Equation with Enzymatic Serum Creatinine** | | | | | |
| **See Appendix 6 of Protocol** | | | | | |
| **Please enter data into the yellow shaded squares (tab between)** | | | | | |
| **Subcalculations will automatically appear in the grey squares** | | | | | |
| **The required values will automatically appear in the red squares** | | | | | |
| CK (Creatine Kinase in units per litre): | | | | | |
| A = 522 x Ln(CK) = | #NUM! | | | | |
| Age (in years): | | | | | |
| B = 34 x Age = | 0 | | | | |
| Y = 4350 + A - B = | #NUM! | | | | |
| Sex (if female enter 1 or if male enter 0): | | | | | |
| Z = 1 - (0.217 x Sex) = | 1 | | | | |
| Weight (in Kg) | | | | | |
| Height (in cm) | | | | | |
| **BSA (Dubois Body Surface Area in square metres) =** | 0 | | | | |
| Numerator = Y x Z x BSA = | #NUM! | | | | |
| ESC (Enzymatic Serum Creatinine in umol per litre): | | | | | |
| **GFR (in ml/min) = Numerator / ESC =** | #NUM! | | | | |
| W = GFR + 25 = | #NUM! | | | | |
| **Carboplatin Dose (mg) = 6 x W =** | #NUM! | | | | |

### WRIGHT EQUATION: JAFFE

$$\text{GFR} = \frac{(4320 - 40 \times \text{Age} + 570 \times \text{Ln(CK)}) \times \text{BSA} \times (1 - 0.15 \times \text{Sex})}{\text{SCr}}$$

| | |
|---|---|
| **BTOG2 Trial** | |
| **Calculation of BSA, GFR and Carboplatin Dose** | |
| **Using Wright Equation with Jaffe Serum Creatinine** | |
| **See Appendix 6 of Protocol** | |
| | |
| **Please enter data into the yellow shaded squares (tab between)** | |
| **Subcalculations will automatically appear in the grey squares** | |
| **The required values will automatically appear in the red squares** | |
| | |
| | |
| CK (Creatine Kinase in units per litre): | |
| A = 570 x Ln(CK) = | #NUM! |
| | |
| Age (in years): | |
| B = 40 x Age = | 0 |
| | |
| Y = 4520 + A - B = | #NUM! |
| | |
| Sex (if female enter 1 or if male enter 0): | |
| Z = 1 - (0.15 x Sex) = | 1 |
| | |
| Weight (in Kg) | |
| Height (in cm) | |
| **BSA (Dubois Body Surface Area in square metres) =** | 0 |
| | |
| Numerator = Y x Z x BSA = | #NUM! |
| | |
| JSC (Jaffe Serum Creatinine in umol per litre): | |
| | |
| **GFR (in ml/min) = Numerator / JSC =** | #NUM! |
| W = GFR + 25 = | #NUM! |
| | |
| **Carboplatin Dose (mg) = 6 x W =** | #NUM! |

Ln(CK) = natural logarithm of creatinine kinase in units of $L^{-1}$

Sex = 1 if female and 0 if male

BSA = Dubois body surface area =0.007184 x weight $^{0.425}$ x height $^{0.725}$

SCr = Serum Creatinine in µmol $L^{-1}$

*Ref:- Wright JG, Boddy AV, Highley M, Fenwick J, McGill A, Calvert AH. Estimation of glomerular filtration rate in cancer patients.Br J Cancer. 2001 Feb;84(4):452-459*

# APPENDIX 7) QUALITY OF LIFE QUESTIONNAIRES

## EORTC QLQ-C30 (version 3)

We are interested in some things about you and your health. Please answer all of the yourself by circling the number that best applies to you. There are no "right" or answers. The information that you provide will remain strictly

Please fill in your initials: | | | |

Your birthdate (Day, Month, Year): | | | |

Today's date (Day, Month, Year): | | | |

| | Not at all | A little | Quite a bit | Very much |
|---|---|---|---|---|
| 1. Do you have any trouble doing strenuous activities, like carrying a heavy shopping bag or a suitcase? | 1 | 2 | 3 | 4 |
| 2. Do you have any trouble taking a long walk? | 1 | 2 | 3 | 4 |
| 3. Do you have any trouble taking a short walk outside of the house? | 1 | 2 | 3 | 4 |
| 4. Do you need to stay in bed or a chair during the day? | 1 | 2 | 3 | 4 |
| 5. Do you need help with eating, dressing, washing yourself or using the toilet? | 1 | 2 | 3 | 4 |

### During the past week:

| | Not at All | A little | Quite a bit | Very much |
|---|---|---|---|---|
| 6. Were you limited in doing either your work or other daily activities? | 1 | 2 | 3 | 4 |
| 7. Were you limited in pursuing your hobbies or other leisure time activities? | 1 | 2 | 3 | 4 |
| 8. Were you short of breath? | 1 | 2 | 3 | 4 |
| 9. Have you had pain? | 1 | 2 | 3 | 4 |
| 10. Did you need to rest? | 1 | 2 | 3 | 4 |
| 11. Have you had trouble sleeping? | 1 | 2 | 3 | 4 |
| 12. Have you felt weak? | 1 | 2 | 3 | 4 |
| 13. Have you lacked appetite? | 1 | 2 | 3 | 4 |
| 14. Have you felt nauseated? | 1 | 2 | 3 | 4 |
| 15. Have you vomited? | 1 | 2 | 3 | 4 |
| 16. Have you been constipated? | 1 | 2 | 3 | 4 |

Please go on to the next page

SPECIMEN

| **During the past week:** | **Not at all** | **A little** | **Quite a bit** | **Very much** |
|---|---|---|---|---|
| 17. Have you had diarrhea? | 1 | 2 | 3 | 4 |
| 18. Were you tired? | 1 | 2 | 3 | 4 |
| 19. Did pain interfere with your daily activities? | 1 | 2 | 3 | 4 |
| 20. Have you had difficulty in concentrating on things, like reading a newspaper or watching television? | 1 | 2 | 3 | 4 |
| 21. Did you feel tense? | 1 | 2 | 3 | 4 |
| 22. Did you worry? | 1 | 2 | 3 | 4 |
| 23. Did you feel irritable? | 1 | 2 | 3 | 4 |
| 24. Did you feel depressed? | 1 | 2 | 3 | 4 |
| 25. Have you had difficulty remembering things? | 1 | 2 | 3 | 4 |
| 26. Has your physical condition or medical treatment interfered with your family life? | 1 | 2 | 3 | 4 |
| 27. Has your physical condition or medical treatment interfered with your social activities? | 1 | 2 | 3 | 4 |
| 28. Has your physical condition or medical treatment caused you financial difficulties? | 1 | 2 | 3 | 4 |

**For the following questions please circle the number between 1 and 7 that best applies to you**

29. How would you rate your overall health during the past week?

| 1 | 2 | 3 | 4 | 5 | 6 | 7 |
|---|---|---|---|---|---|---|
| Very poor | | | | | | Excellent |

30. How would you rate your overall quality of life during the past week?

| 1 | 2 | 3 | 4 | 5 | 6 | 7 |
|---|---|---|---|---|---|---|
| Very poor | | | | | | Excellent |

SPECIMEN

© Copyright 1995 EORTC Quality of Life Study Group. All rights reserved. Version 3.0

# EORTC QLQ-LC13

Patients sometimes report that they have the following symptoms or problems. Please indicate the extent to which you have experienced these symptoms or problems during the past week. Please answer by circling the number that best applies to you.

| **During the past week:** | **Not at all** | **A little** | **Quite a bit** | **Very much** |
|---|---|---|---|---|
| 1. How much did you cough? | 1 | 2 | 3 | 4 |
| 2. Did you cough blood? | 1 | 2 | 3 | 4 |
| 3. Were you short of breath when you rested? | 1 | 2 | 3 | 4 |
| 4. Were you short of breath when you walked? | 1 | 2 | 3 | 4 |
| 5. Were you short of breath when you climbed stairs? | 1 | 2 | 3 | 4 |
| 6. Have you had a sore mouth or tongue? | 1 | 2 | 3 | 4 |
| 7. Have you had trouble swallowing? | 1 | 2 | 3 | 4 |
| 8. Have you had tingling hands or feet? | 1 | 2 | 3 | 4 |
| 9. Have you had hair loss? | 1 | 2 | 3 | 4 |
| 10. Have you had pain in your chest? | 1 | 2 | 3 | 4 |
| 11. Have you had pain in your arm or shoulder? | 1 | 2 | 3 | 4 |
| 12. Have you had pain in other parts of your body? | 1 | 2 | 3 | 4 |
| If yes, where? ............................................... | | | | |
| 13. Did you take any medicine for pain? Yes No | | | | |
| If yes, did it help? | 1 | 2 | 3 | 4 |

SPECIMEN

©Copyright 1994 EORTC Quality of Life Study Group. Version 1.0. All rights reserved.

# EUROQOL EQ-5D HEALTH QUESTIONNAIRE

**By placing a tick in one box in each group below, please indicate which statements best describe your health state today**

**Mobility**

I have no problems in walking about ☐

I have some problems in walking about ☐

I am confined to bed ☐

**Self-Care**

I have no problems with self-care ☐

I have some problems washing or dressing myself ☐

I am unable to wash or dress myself ☐

**Usual Activities** *(e.g. work, study, housework, family or leisure activities)*

I have no problems with performing my usual activities ☐

I have some problems with performing my usual activities ☐

I am unable to perform my usual activities ☐

**Pain/Discomfort**

I have no pain or discomfort ☐

I have moderate pain or discomfort ☐

I have extreme pain or discomfort ☐

**Anxiety/Depression**

I am not anxious or depressed ☐

I am moderately anxious or depressed ☐

I am extremely anxious or depressed ☐

SPECIMEN

To help people say how good or bad a health state is, we have drawn a scale (rather like a thermometer) on which the best state you can imagine is marked 100 and the worst state you can imagine is marked 0.

We would like you to indicate on this scale how good or bad your own health is today, in your opinion. Please do this by drawing a line from the box below to whichever point on the scale indicates how good or bad your health is today.

**Your own heatlh state today**

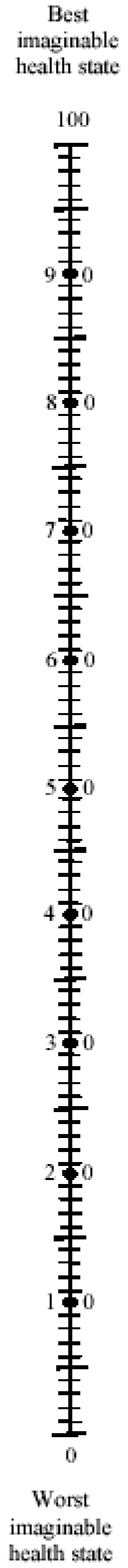


SPECIMEN

# APPENDIX 8) RECIST CRITERIA

The following contains excerpts from the recently published RECIST criteria. For more information, a full copy can be seen at http://www.eortc.be. *Ref. P. Therasse, S. A. Arbuck, E. A. Eisenhauer et al., New Guidelines to evaluate the response to treatment in solid tumors. Journal Of the National cancer institute Vol 92, No 3, Feb2, p 205.*

The selected sections are named as in the full RECIST document

## *Section 2* MEASURABILITY OF TUMOUR LESIONS AT BASELINE

### *2.1* Definitions

At baseline, tumour lesions will be categorised as follows: measurable (lesions that can be accurately measured in at least one dimension [longest diameter to be recorded] as 20 mm with conventional techniques or as 10 mm with spiral CT scan [*see* section 2.2]) or nonmeasurable (all other lesions, including small lesions [longest diameter <20 mm with conventional techniques or <10 mm with spiral CT scan] and truly nonmeasurable lesions).

The term "evaluable" in reference to measurability is not recommended and will not be used because it does not provide additional meaning or accuracy.
All measurements should be recorded in metric notation by use of a ruler or calipers. All baseline evaluations should be performed as closely as possible to the beginning of treatment and never more than 4 weeks before the beginning of treatment.

Lesions considered to be truly nonmeasurable include the following: bone lesions, leptomeningeal disease, ascites, pleural/pericardial effusion, inflammatory breast disease, lymphangitis cutis/pulmonis, abdominal masses that are not confirmed and followed by imaging techniques, and cystic lesions.
(*Note:* Tumour lesions that are situated in a previously irradiated area might or might not be considered measurable, and the conditions under which such lesions should be considered must be defined in the protocol when appropriate.)

## *Section 3* TUMOUR RESPONSE EVALUATION

### *3.1* Baseline evaluation

#### *3.1.1* Assessment of overall tumour burden and measurable disease

To assess objective response, it is necessary to estimate the overall tumour burden at baseline to which subsequent measurements will be compared. Only patients with measurable disease at baseline should be included in protocols where objective tumour response is the primary end point. Measurable disease is defined by the presence of at least one measurable lesion (as defined in section 2.1). If the measurable disease is restricted to a solitary lesion, its neoplastic nature should be confirmed by cytology/histology.

#### *3.1.2* Baseline documentation of "target" and "nontarget" lesions

All measurable lesions up to a maximum of five lesions per organ and 10 lesions in total, representative of all involved organs, should be identified as target lesions and recorded and

measured at baseline. Target lesions should be selected on the basis of their size (those with the longest diameter) and their suitability for accurate repeated measurements (either by imaging techniques or clinically). A sum of the longest diameter for all target lesions will be calculated and reported as the baseline sum longest diameter. The baseline sum longest diameter will be used as the reference by which to characterise the objective tumour response**.**

All other lesions (or sites of disease) should be identified as nontarget lesions and should also be recorded at baseline. Measurements of these lesions are not required, but the presence or absence of each should be noted throughout follow-up.

### *3.2* Response criteria

#### *3.2.1* Evaluation of target lesions

This section provides the definitions of the criteria used to determine objective tumour response for target lesions. The criteria have been adapted from the original WHO Handbook, taking into account the measurement of the longest diameter only for all target lesions: complete response—the disappearance of all target lesions; partial response—at least a 30% decrease in the sum of the longest diameter of target lesions, taking as reference the baseline sum longest diameter; progressive disease—at least a 20% increase in the sum of the longest diameter of target lesions, taking as reference the smallest sum longest diameter recorded since the treatment started or the appearance of one or more new lesions; stable disease—neither sufficient shrinkage to qualify for partial response nor sufficient increase to qualify for progressive disease, taking as reference the smallest sum longest diameter since the treatment started.

#### *3.2.2* Evaluation of nontarget lesions

This section provides the definitions of the criteria used to determine the objective tumour response for nontarget lesions: complete response—the disappearance of all nontarget lesions and normalisation of tumour marker level; incomplete response/stable disease—the persistence of one or more nontarget lesion(s) and/or the maintenance of tumour marker level above the normal limits; and progressive disease—the appearance of one or more new lesions and/or unequivocal progression of existing nontarget lesions.

(*Note:* Although a clear progression of "nontarget" lesions only is exceptional, in such circumstances, the opinion of the treating physician should prevail and the progression status should be confirmed later by the review panel [or study chair]).

#### *3.2.3* Evaluation of best overall response

The best overall response is the best response recorded from the start of treatment until disease progression/recurrence (taking as reference for progressive disease the smallest measurements recorded since the treatment started). In general, the patient's best response assignment will depend on the achievement of both measurement and confirmation criteria (*see* section 3.3.1). Table provides overall responses for all possible combinations of tumour responses in target and nontarget lesions with or without the appearance of new lesions.
(*Notes:*

- Patients with a global deterioration of health status requiring discontinuation of treatment without objective evidence of disease progression at that time should be classified as having "symptomatic deterioration." Every effort should be made to document the objective disease progression, even after discontinuation of treatment.

- Conditions that may define early progression, early death, and inevaluability are study specific and should be clearly defined in each protocol (depending on treatment duration and treatment periodicity).
- In some circumstances, it may be difficult to distinguish residual disease from normal tissue. When the evaluation of complete response depends on this determination, it is recommended that the residual lesion be investigated (fine-needle aspiration/biopsy) before confirming the complete response status.)

**Table 1.** Overall responses for all possible combinations of tumour responses in target and nontarget lesions with or without the appearance of new lesions

| **Target lesions** | **Nontarget lesions** | **New lesions** | **Overall response** |
|---|---|---|---|
| CR | CR | No | CR |
| CR | Incomplete response/SD | No | PR |
| PR | Non-PD | No | PR |
| SD | Non-PD | No | SD |
| PD | Any | Yes or no | PD |
| Any | PD | Yes or no | PD |
| Any | Any | Yes | PD |

CR = complete response; PR = partial response; SD = stable disease; and PD = progressive disease. *See* text for more details.

## *Section 5* REPORTING OF RESULTS

All patients included in the study must be assessed for response to treatment, even if there are major protocol treatment deviations or if they are ineligible. Each patient will be assigned one of the following categories: 1) complete response, 2) partial response**,** 3) stable disease, 4) progressive disease, 5) early death from malignant disease, 6) early death from toxicity, 7) early death because of other cause, or 9) unknown (not assessable, insufficient data). (*Note:* By arbitrary convention, category 9 usually designates the "unknown" status of any type of data in a clinical database.)

All of the patients who met the eligibility criteria should be included in the main analysis of the response rate. Patients in response categories 4-9 should be considered as failing to respond to treatment (disease progression). Thus, an incorrect treatment schedule or drug administration does not result in exclusion from the analysis of the response rate. Precise definitions for categories 4-9 will be protocol specific.

All conclusions should be based on all eligible patients.

Subanalyses may then be performed on the basis of a subset of patients, excluding those for whom major protocol deviations have been identified (e.g. early death due to other reasons, early discontinuation of treatment, major protocol violations, etc). However, these subanalyses may not serve as the basis for drawing conclusions concerning treatment efficacy, and the reasons for excluding patients from the analysis should be clearly reported. The 95% confidence intervals should be provided.

# APPENDIX 9) ABBREVIATIONS

| | |
|---|---|
| ADR | Adverse Drug Reaction |
| AE | Adverse Event |
| ALT | Alanine Aminotransferase |
| ANC | Absolute Neutrophil Count |
| AST | Asparate Aminotransferase |
| AUC | Area Under Curve |
| BTOG | British Thoracic Oncology Group |
| CI | Confidence Interval |
| CRCTU | Cancer Research UK Clinical Trials Unit |
| CRF | Case Report Form |
| DSMC | Independent Data and Safety Monitoring Committee |
| ECOG | Eastern Cooperative Oncology Group |
| EORTC | European Organization for Research and Treatment of Cancer |
| GCP | Good Clinical Practice |
| MHRA | Medicines and Healthcare products Regulatory Agency |
| MIC | Mitomycin, Ifosfamide and Cisplatin |
| MVP | Mitomycin, Vinblastine and Cisplatin |
| NSCLC | Non-Small Cell Lung Cancer |
| QALY | Quality-Adjusted Life Years |
| QLQ | Quality of Life Questionnaire |
| RECIST | Response Evaluation Criteria in Solid Tumours |
| SAE | Serious Adverse Event |
| SCLC | Small Cell Lung Cancer |
| SSAR | Suspected serious adverse reaction |
| SUSAR | Suspected unexpected serious adverse reaction |
| ULN | Upper Limits of Normal |
| WBC | White Blood Cell Count |
| WHO | World Health Organization |

**BTOG2 Study Office**
Cancer Research UK Clinical Trials Unit
Institute for Cancer Studies
The University of Birmingham
Edgbaston
Birmingham B15 2TT
Tel 0121 414 6425
Fax 0121 414 2230
Email BTOG2@trials.bham.ac.uk
Website: www.BTOG2.bham.ac.uk
